# GABA Neuronal Deletion of *Shank3* Exons 14–16 in Mice Suppresses Striatal Excitatory Synaptic Input and Induces Social and Locomotor Abnormalities

**DOI:** 10.3389/fncel.2018.00341

**Published:** 2018-10-09

**Authors:** Taesun Yoo, Heejin Cho, Jiseok Lee, Haram Park, Ye-Eun Yoo, Esther Yang, Jin Yong Kim, Hyun Kim, Eunjoon Kim

**Affiliations:** ^1^Department of Biological Sciences, Korea Advanced Institute for Science and Technology (KAIST), Daejeon, South Korea; ^2^Center for Synaptic Brain Dysfunctions, Institute for Basic Science (IBS), Daejeon, South Korea; ^3^Department of Anatomy and Division of Brain Korea 21, Biomedical Science, College of Medicie, Korea University, Seoul, South Korea

**Keywords:** autism, Phelan-McDermid syndrome, Shank3, striatum, social interaction, repetitive behavior

## Abstract

Shank3 is an excitatory postsynaptic scaffolding protein implicated in multiple brain disorders, including autism spectrum disorders (ASD) and Phelan-McDermid syndrome (PMS). Although previous neurobiological studies on Shank3 and *Shank3*-mutant mice have revealed diverse roles of Shank3 in the regulation of synaptic, neuronal and brain functions, whether Shank3 expression in specific cell types distinctly contributes to mouse phenotypes remains largely unclear. In the present study, we generated two *Shank3*-mutant mouse lines (exons 14–16) carrying global and GABA neuron-specific deletions and characterized their electrophysiological and behavioral phenotypes. These mouse lines show similar decreases in excitatory synaptic input onto dorsolateral striatal neurons. In addition, the abnormal social and locomotor behaviors observed in global *Shank3*-mutant mice are strongly mimicked by GABA neuron-specific *Shank3*-mutant mice, whereas the repetitive and anxiety-like behaviors are only partially mimicked. These results suggest that GABAergic *Shank3* (exons 14–16) deletion has strong influences on striatal excitatory synaptic transmission and social and locomotor behaviors in mice.

## Introduction

Shank represents a family of postsynaptic scaffolding proteins with three known members: Shank1/ProSAP3, Shank2/ProSAP1 and Shank3/ProSAP2 (Sheng and Kim, 2000; Sheng and Sala, 2001; Boeckers et al., 2002; Sheng and Hoogenraad, 2007; Grabrucker et al., 2011; Sheng and Kim, 2011; Jiang and Ehlers, 2013; Sala et al., 2015; Monteiro and Feng, 2017; Mossa et al., 2017). Shank proteins interact with many other synaptic proteins and are known to regulate excitatory synapse assembly as well as excitatory synaptic transmission and plasticity.

Mutations of *SHANK3* (Boeckers et al., 1999; Lim et al., 1999; Naisbitt et al., 1999; Tu et al., 1999) have been implicated in diverse brain disorders, including autism spectrum disorders (ASD), neurological and psychiatric symptoms of Phelan-McDermid syndrome (PMS), schizophrenia, intellectual disability and mania (Phelan et al., 1993; Bonaglia et al., 2001; Wilson et al., 2003; Durand et al., 2007; Moessner et al., 2007; Gauthier et al., 2010; Bonaglia et al., 2011; Hamdan et al., 2011; Leblond et al., 2012; Boccuto et al., 2013; Han et al., 2013; Guilmatre et al., 2014; Leblond et al., 2014; Cochoy et al., 2015; De Rubeis et al., 2018).

A number of *Shank3*-mutant mouse lines have been generated and characterized in an effort to understand the *in vivo* functions of Shank3 and identify important mechanisms underlying *Shank3-related* brain disorders (Bozdagi et al., 2010; Peca et al., 2011; Wang et al., 2011; Schmeisser et al., 2012; Yang et al., 2012; Han et al., 2013; Kouser et al., 2013; Lee et al., 2015; Speed et al., 2015; Jaramillo et al., 2016; Mei et al., 2016; Wang et al., 2016; Zhou et al., 2016; Jaramillo et al., 2017; Vicidomini et al., 2017; Bey et al., 2018; Qin et al., 2018).

Given that Shank3 is an important component of excitatory synapses (Boeckers et al., 1999; Lim et al., 1999; Naisbitt et al., 1999; Tu et al., 1999), and that the imbalance of excitation and inhibition (E/I) at synaptic and neuronal levels has been implicated in ASD (Yizhar et al., 2011; Nelson and Valakh, 2015; Lee E. et al., 2017), Shank3 dysfunctions may have significant influences on E/I imbalances associated with ASD. Importantly, however, because *Shank3* is expressed in both excitatory and inhibitory neurons (Han et al., 2013), the consequences of *Shank3* mutations in mixed neuronal populations are not easy to predict and should be assessed by direct cell type-specific *Shank3* deletion *in vivo* for better understanding of related brain regions, cell types, and neural circuits. In further support of the importance of *Shank3* expression in GABAergic neurons, *Shank3* is highly expressed in the striatum (Peca et al., 2011), a brain region enriched with GABAergic neurons and known to be associated with various brain functions as well as neurological and psychiatric disorders (Balleine et al., 2007; Kreitzer and Malenka, 2008; Grueter et al., 2012; Báez-Mendoza and Schultz, 2013). In addition, GABAergic neurons in the striatum have dendritic spines where Shank3 may play important roles in the regulation of spinogenesis and axospinous synapse functions (Harris and Weinberg, 2012; O’Rourke et al., 2012).

To this end, we attempted a GABA neuron-specific deletion of *Shank3* exons 14–16, which encodes the PDZ domain known to interact with many synaptic proteins, including GKAP/SAPAP (Kim and Sheng, 2004; Sheng and Kim, 2011), using the Viaat-Cre mouse line that drives Cre recombinase expression in widespread GABAergic neurons (Chao et al., 2010). The electrophysiological and behavioral phenotypes of these mice were compared with those from mice carrying a global *Shank3* deletion (exons 14–16). We found that GABA neuron-specific *Shank3* deletion induces a strong reduction in excitatory synaptic input onto dorsolateral striatal neurons and abnormal social and locomotor behaviors, while having moderate effects on repetitive and anxiety-like behaviors.

## Materials and Methods

### Animals

Mice carrying a deletion of exons 14–16 of the *Shank3* gene flanked by LoxP sites were designed and generated by Biocytogen. The EGFP+ Neo cassette was eliminated by crossing these mice with *protamine*-Flp mic. EGFP+ Neo cassette-deleted *Shank3*^flox/+^ mice were crossed with *protamine-Cre* mice, and the resulting mice were then crossed with wild-type (WT) mice to introduce the *Shank3*^Δ14–16^ allele. Experimental *Shank3*^Δ14–16^ global knockout mice were obtained by heterozygous mating (*Shank3*^Δ^14–16/+ × *Shank3*^Δ14–16/+^). To generate *Shank3*^Δ14–16^ cell type-specific conditional knockout (cKO) mice in which *Shank3* is knocked out in Viaat (vesicular inhibitory amino acid transporter)-expressing GABAergic neurons (*Viaat-Cre;Shank3*^fl/fl^ mice), homozygous *Shank3*^flox/flox^ female mice were crossed with double-heterozygous *Viaat-Cre;Shank3*^flox/+^ male mice. The control group for the cKO mouse was Cre-negative *Shank3*^flox/flox^ littermates. *Viaat-Cre*, *protamine-Flp* and *protamine-Cre* mouse lines used in this study were maintained in a C57BL/6J genetic background for more than five generations, a breeding strategy that allowed us to compare all global and *Viaat-Cre* mouse line in the same pure C57BL/6J background. All mice were bred and maintained at the mouse facility of Korea Advanced Institute of Science and Technology (KAIST) according to Animal Research Requirements of KAIST, and all experimental procedures were approved by the Committee of Animal Research at KAIST (KA2016-30). All animals were fed *ad libitum* and housed under the 12 h light/dark cycle (light phase during 1:00 am to 1:00 pm). Polymerase chain reaction (PCR) genotyping of conventional knockout mice was performed using the following primers: for WT allele (276 bp): 5’-GGG TTC CTA TGA CAG CCT CA-3’ and 5’-TTC TGC AGG ATA GCC ACC TT-3’; for deletion (del) allele (1,159 bp): 5’-GGG TTC CTA TGA CAG CCT CA-3’ and 5’-AGC TCA GCC GTC ATG GAC-3’. Genotypes of *Viaat-Cre;Shank3*^fl/fl^ mice were determined by PCR using the following primers: for floxed (478 bp) or WT allele (276 bp): 5’-GGG TTC CTA TGA CAG CCT CA-3’ and 5’-TTC TGC AGG ATA GCC ACC TT-3’; for Viaat-Cre allele (272 bp): 5’-GTG TTG CCG CGC CAT CTG C-3’ and 5’-CAC CAT TGC CCC TGT TTC ACT ATC-3’. Only male mice were used for behavioral and electrophysiological experiments. Both male and female were used for biochemical experiments.

### Fluorescent *in situ* Hybridization (FISH)

In brief, frozen sections (14 μm thick) were cut coronally through the cortex and striatum formation. Sections were thaw-mounted onto Superfrost Plus Microscope Slides (Fisher Scientific #12-550-15). The sections were fixed in 4% formaldehyde for 10 min, dehydrated in increasing concentrations of ethanol for 5 min, and finally air-dried. Tissues were then pretreated for protease digestion for 10 min at room temperature. Probe hybridization and amplification were performed at 40°C using HybEZ hybridization oven (Advanced Cell Diagnostics, Hayward, CA, USA). The probes used in this study were three synthetic oligonucleotides complementary to the nucleotide (nt) sequence 1488–2346 of Mm-Shank3, nt 62–3113 of Mm-Gad1-C3, nt 552–1506 of Mm-Gad2-C2, nt 464–1415 of Mm-Slc17a7/Vglut1-C2, and nt 1986–2998 of Mm-Slc17a6/Vglut2-C3 (Advanced Cell Diagnostics, Hayward, CA, USA). The labeled probes were conjugated to Alexa Fluor 488, Atto 550, and Atto 647. The sections were hybridized with the labeled probe mixture at 40°C for 2 h per slide. Unbound hybridization probes were removed by washing the sections three times with 1× wash buffer at room temperature for 2 min. Following steps for signal amplification included incubations at 40°C with Amplifier 1-FL for 30 min, with Amplifier 2-FL for 15 min, with Amplifier 3-FL for 30 min and with Amplifier 4 Alt B-FL for 15 min. Each amplifier solution was removed by washing with 1× wash buffer at room temperature for 2 min. The slides were viewed, analyzed and photographed using TCS SP8 Dichroic/CS (Leica), and the ImageJ program (NIH) was used to analyze the images.

### Brain Lysates

Brains from *Shank3*^Δ14–16^ mice and their WT littermates (13 weeks; male), and those from *Viaat-Cre;Shank3*^fl/fl^ mice and their WT littermates (12 weeks; female), were extracted and dissected on ice into cortex, thalamus, striatum and hippocampus, followed by homogenization with ice-cold homogenization buffer (0.32 M sucrose, 10 mM HEPES, pH 7.4, 2 mM EDTA, pH 8.0, 2 mM EGTA, pH8.0, protease inhibitors, phosphatase inhibitors). Total lysates were prepared by boiling with β-mercaptoethanol directly after homogenization.

### Western Blot

Total brain lysates separated in electrophoresis and transferred to a nitrocellulose membrane were incubated with primary antibodies to Shank3 (#2036 guinea pig polyclonal antibodies raised against aa 1289–1318 of the mouse Shank3 protein, 1:500; Lee et al., 2015) and α-tubulin (Sigma T5168; 1:1,000) at 4°C overnight. Fluorescent secondary antibody signals were detected using *Odyssey*^®^ *Fc Dual Mode Imaging System*.

### Rat Neuron Culture, Immunocytochemistry and Imaging

Primary hippocampal neuronal cultures were prepared from Sprague-Dawley rats at E18 as described previously (Goslin and Banker, 1991). Dissociated neurons were plated in coverslips coated with poly-L-lysine and laminin, and grown in neurobasal media supplemented with B27 (Invitrogen), 0.5 mM glutamax (Invitrogen) and 12.5 μM glutamate (plating media) in a 10% CO_2_ incubator. After, this plating media and maintained media were replaced with feeding media (same as plating media only except for glutamate) every week. For immunocytochemistry, cultured neurons (at days *in vitro* or DIV 15) were fixed with 1% paraformaldehyde/1% sucrose (5 min) and methanol (5 min), permeabilized with 0.1% gelatin, 0.3% Triton X-100, 450 mM NaCl in phosphate buffered saline (PBS), and immunostained with primary antibodies against Shank3 (Santa Cruz H-160, 1:200) and GAD67 (Abcam ab26116, 1:200), and FITC-, and Alexa594-conjugated secondary antibodies (Jackson ImmunoResearch). The images were acquired using a confocal microscope (LSM780, Carl Zeiss) with a ×63 objective lens. The Z-stacked images were converted to maximal projection.

### Electrophysiology

Mice at P28–35 (for dorsolateral striatum mEPSC and mIPSC) were anesthetized with diethyl ether. Mouse brain sections (300 μm) were sectioned in ice-cold dissection buffer containing (in mM) 212 sucrose, 25 NaHCO_3_, 10 D-glucose, 2 Na-pyruvate, 1.25 ascorbic acid, 1.25 NaH_2_PO_4_, 5 KCl, 3.5 MgSO_4_ and 0.5 CaCl_2_ bubbled with 95% O_2_ and 5% CO_2_ gases using Leica VT 1,200 vibratome. The slices were recovered for 30 min and maintained in artificial cerebrospinal fluid (ACSF) at 32°C (in mM: 124 NaCl, 25 NaHCO_3_, 10 Glucose, 2.5 KCl, 1 NaH_2_PO_4_, 2.5 CaCl_2_, 1.3 MgSO_4_ oxygenated with 95% O_2_ and 5% CO_2_ gases). All recordings were performed after recovery for additional 30 min at room temperature. During all recordings, brain slices were maintained in a submerge-type recording chamber perfused with 26.5–28°C ACSF (2 ml min^−1^). Recording and stimulus glass pipettes from borosilicate glass capillaries (Harvard Apparatus) were pulled using an electrode puller (Narishige). All electric responses were amplified and filtered at 2 kHz (Multiclamp 700B, Molecular Devices) and then digitized at 10 kHz (Digidata 1550, Molecular Devices). For whole-cell patch recordings in the dorsolateral striatum, a recording pipette (2.5–3.5 MΩ) was filled with the internal solution (in mM: 100 CsMeSO_4_, 10 TEA-Cl, 8 NaCl, 10 HEPES, 5 QX-314-Cl, 2 Mg-ATP, 0.3 Na-GTP and 10 EGTA for mEPSCs; 115 CsCl, 10 EGTA, 8 NaCl, 10 TEACl, 10 HEPES, 4 Mg-ATP, 0.3 Na-GTP, 5 QX-314 for mIPSCs) adjusted to pH 7.35 and 285 mOsm. To measure mEPSCs and mIPSCs, dorsolateral striatal MSN neurons were voltage-clamped at −70 mV. For mEPSCs and mIPSCs, picrotoxin (60 μM) and NBQX (10 μM) + APV (50 μM) were added to ACSF with TTX (1 μM), respectively. Responses were recorded for 2 min after maintaining stable baseline for 5 min. MSNs in the dorsal striatum were identified by the soma size (8–12 μm) and basic membrane properties (cell capacitance >100 pF and input resistance >160 MOhm, as reported previously (Cepeda et al., 1998, 2008; Gertler et al., 2008).

### Behavioral Assays

Before behavioral experiments, all mice were handled for 10 min per day for 3 days. All behavioral assays were proceeded after 30 min habituation in a dark booth. All tested mice were 2–7 months male mice. The order of behavioral tests was designed in a way to minimize stress in animals. The behavioral tests for global *Shank3*^Δ14–16^ and *Viaat-Cre;Shank3*^Δ14–16^ mice were performed in the orders described in Supplementary Table S1.

### Three-Chamber Test

Social approach was measured using the three-chambered test (Moy et al., 2004; Nadler et al., 2004; Silverman et al., 2010). The apparatus is a white acrylic box (60 × 40 × 20 cm) divided into three chambers. The illumination condition was ~10 lux for global *Shank3*^Δ14–16^ mice and 70–80 lux for *Viaat-Cre;Shank3*^Δ14–16^ mice. We used a dim light condition (~10 lux) for global *Shank3*^Δ14–16^ mice because a brighter light condition (~70–80 lux) did not yield optimal results in WT mice. Both left and right side chambers contained a cage in the upper or lower corner for an object or a stranger mouse. Experimental mice were isolated in a single cage for 3 days prior to the test, whereas unfamiliar stranger mice (129S1/SvlmJ strain) were group-housed (5–7 mice/cage). All stranger mice were age-matched males and were habituated to a corner cage during the previous day (30 min). The test consisted of three phases: empty-empty (habituation), stranger1-object (S1-O) and stranger1-stranger2 (S1-S2). In the first (habituation) phase, a test mouse was placed in the center area of the three-chambered apparatus, and allowed to freely explore the whole apparatus for 10 min. The mouse was then gently guided to the center chamber while an inanimate blue cylindrical object (O) and a WT stranger mouse, termed stranger 1 (S1), were placed in the two corner cages. The positions of object (O) and S1 were alternated between tests to prevent side preference. In the S1-O phase, the test mouse was allowed to explore the stranger mouse or the object freely for 10 min. Before the third S1-S2 phase, the subject mouse was again gently guided to the center chamber while the object was replaced with a new WT stranger mouse, termed stranger 2 (S2). The subject mouse again was allowed to freely explore all three chambers and interact with both stranger mice for 10 min. The duration of sniffing, defined as positioning of the nose of the test mouse within 2.5 cm from a cage, was measured using Ethovision XT10 (Noldus) software.

### Direct Social Interaction Test

Each individual mouse spent 10 min in a gray box (30 × 30 × 30 cm; ~25–30 lux) for two consecutive days for habituation. On day 3, pairs of mice of the same genotype (originally housed separately) were placed in the test box for 10 min. All mice were isolated for 3 days prior to the experimental day. Time spent in nose-to-nose interaction, following, and total interaction were measured manually in a blinded manner. Nose-to-nose interaction was defined as sniffing the head part of the other mouse. Following included the behavior of a mouse following the other mouse as well as nose-to-tail sniffing. Total interaction included nose-to-nose interaction, following, body contact, allo-grooming and mounting.

### Courtship Ultrasonic Vocalization

Adult subject male mice were isolated in their home cage for 3 days before the test, whereas age-matched intruder female mice were group-housed (6–7 mice/cage). We did not measure female estrous cycles, assuming that group housing may synchronize the cycles. Basal ultrasonic vocalizations (USVs) of an isolated male mouse in its home cage under a light condition of ~60 lux in a soundproof chamber were recorded for 5 min in the absence of a female intruder. Next, a randomly chosen stranger C57BL/6J female mouse was introduced into the cage, and female-induced courtship USVs were recorded for 5 min during free interaction between males and females. Avisoft SASLab Pro software was used to automatically analyze the number of USV calls, latency to first call, and total duration of calls from recorded USV files. Signals were filtered from 1 Hz to 100 kHz and digitized with a sampling frequency of 250 kHz, 16 bits per sample (Avisoft UltraSoundGate 116H). To generate spectrograms, the following parameters were used (FFT length: 256, frame size: 100, window: FlatTop, overlap: 75%), resulting in a frequency resolution of 977 Hz and a temporal resolution of 0.256 msec. Frequencies lower than 25 kHz were filtered out to reduce background white noises.

### Repetitive Behavior and Self-Grooming Test

Each mouse was placed in a fresh home cage (~60–70 lux) with bedding and recorded for 20 min. The last 10 min was analyzed manually to measure times spent in self-grooming and digging behavior. Self-grooming behavior was defined as stroking or scratching of its body or face, or licking its body parts. Digging was defined as the behavior of scattering bedding using its head and forelimbs. To further analyze self-grooming behavior, mice were placed in an empty home cage without bedding and were recorded for 20 min. Time spent in self-grooming behavior was counted manually during the last 10 min in a blind manner.

### Laboras Test (Long-Term Monitoring)

Each mouse was placed in a single cage and recorded for 96 consecutive hours from the start of the night cycle. Illumination condition during light-on periods was ~60 lux. Basal activities (locomotion, climbing, rearing, grooming, eating and drinking) were recorded and automatically analyzed by the Laboratory Animal Behavior Observation Registration and Analysis System (LABORAS, Metris). Laboras results were not validated by own manual analyses, given the availability of previous validation results (Van de Weerd et al., 2001; Quinn et al., 2003, 2006; Dere et al., 2015). Mouse movements during the whole 4-day period were used for quantification, except for other behaviors, for which movements during light-off periods were used for more clear results.

### Open-Field Test

Mice were put in the center of a white acrylic box (40 × 40 × 40 cm), and their locomotion was recorded with a video camera for 1 h. The illumination of the open field was 90–100 lux. The recorded video was analyzed using Ethovision XT10 software (Noldus). The center zone was defined as an area with 4 × 4 squares when the whole-field was 6 × 6 squares.

### Elevated Plus-Maze Test

The maze was elevated to a height of 75 cm from the floor, with two open arms (30 × 6 cm, ~180 lux) and two closed arms (30 × 6 cm, ~20 lux). Mice were introduced onto the center of the apparatus with their head toward the open arms and allowed to freely explore the environment for 8 min. Amounts of time spent in open or closed arms and number of transitions were measured by Ethovision XT10 software (Noldus).

### Light-Dark Test

The light-dark apparatus was divided into light and dark chambers (21 × 29 × 20 cm, 700 lux, light chamber; 21 × 13 × 20 cm, ~5 lux, dark chamber) separated by an entrance in the middle wall (5 × 8 cm). Mice were introduced in the light chamber with their head toward the opposite side of the dark chamber and allowed to freely explore the apparatus for 10 min. Amounts of time spent in light and dark chambers and number of transitions were analyzed by Ethovision XT10 software (Noldus).

### Statistical Analysis

Statistical analyses were performed using GraphPad Prism 5 software. Details of statistical analyses and results are presented in Supplementary Table S2. The normality of the data distribution was determined using the D’Agostino and Pearson omnibus normality test, followed by Student’s *t*-test (in the case of normal distribution) and Mann- Whitney U test (in the case of non-normal distribution). If, sample is dependent each other, paired *t*-test (in the case of normal distribution), and Wilcoxon signed rank test (in the case of non-normal distribution). Repeated-measures of two-way ANOVA and subsequent Bonferroni *post hoc* multiple comparison tests, performed only when there are significant interactions, were used for the time-varying analysis of open-field test and Laboras test. If a single value makes the data distribution as non-normal and is detected as significant outlier (*P < 0.05) under the Grubb’s test, we removed the data as outliers. One sample *t*-test was used for the analysis of western blot data. The statistical significance of values are indicated in the figure panels as follows: *P < 0.05, **P < 0.01, ***P < 0.001, nd, not detectable and ns, not significant.

## Results

### Expression of *Shank3* in Both Glutamatergic and GABAergic Neurons

**Figure 1:**
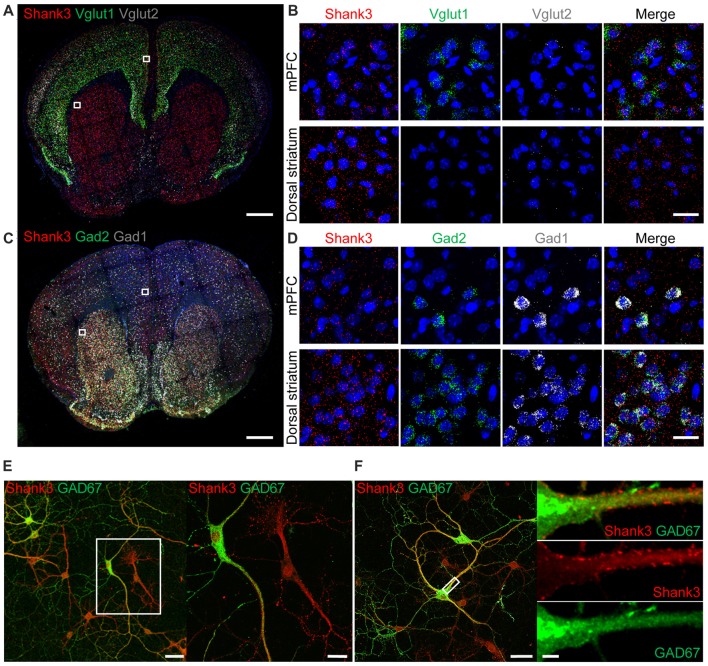
Expression of *Shank3* in both glutamatergic and GABAergic neurons. **(A,B)** Detection of *Shank3* mRNA in *Vglut1/2* mRNA-positive glutamatergic neurons in the prelimbic region of the medial prefrontal cortex (mPFC) in mice (P56) by double-immunofluorescence *in situ* hybridization. Note that *Vglut2* mRNA signals in the mPFC were weaker than those of *Vglut1*, and that, in the dorsolateral striatum, *Vglut1/2* mRNA signals are very weak or absent. Scale bar, 0.5 mm, 20 μm. **(C,D)** Detection of *Shank3* mRNA in Gad1/2-positive GABAergic neurons in the prelimbic region of the mPFC and the dorsolateral region of the striatum of mice (P56) by double-immunofluorescence *in situ* hybridization. Scale bar, 0.5 mm, 20 μm. **(E,F)** Detection of Shank3 proteins in GAD67-positive GABAergic neurons in cultured rat hippocampal neurons at 15 days *in vitro* (DIV 15), as shown by double immunofluorescence staining for Shank3 and GAD67 (encoded by *Gad1*). Scale bar, 50 μm, 20 μm, 50 μm, 5 μm.

To explore the contributions of *Shank3* expression in excitatory and inhibitory neurons to synaptic functions and behaviors in mice, we first tested whether *Shank3* is expressed in glutamatergic and GABAergic neurons using fluorescence *in situ* hybridization (FISH). *Shank3* *in situ* signals were present in Vglut1- and Vglut2-positive glutamatergic neurons in brain regions including the medial prefrontal cortex (mPFC; Figures 1A,B), indicative of *Shank3* expression in glutamatergic excitatory neurons. *Shank3* signals were also present in Gad1- and Gad2-positive GABAergic neurons in brain regions including the mPFC and the dorsolateral region of the striatum (Figures 1C,D). These results suggest that *Shank3* mRNA is expressed in both glutamatergic and GABAergic neurons. *Shank3* mRNA signals outside of DAPI-labeled nuclei or neighboring cell body regions may represent dendritic (rather than somatic) *Shank3* mRNA, as previously reported (Epstein et al., 2014).

To further characterize Shank3 expression in GABAergic neurons, we immunostained for Shank3 protein in GABAergic neurons in cultured rat hippocampal neurons. Shank3 signals were detected in dendrites of both GAD67 (encoded by Gad1)-positive GABAergic neurons and GAD67-negative cells (Figure 1E). In addition, punctate Shank3 signals were observed at shaft excitatory synapses on dendrites of GAD67-positive GABAergic neurons (Figure 1F). These results, together with the previously reported positive expression of EGFP-tagged Shank3 in GAD-6–positive GABAergic neurons (Han et al., 2013), suggest that Shank3 is expressed in both glutamatergic and GABAergic neurons.

### Generation and Characterization of Global *Shank3*^Δ14–16^ and *Viaat-Cre;Shank3*^Δ14–16^ Mice

**Figure 2:**
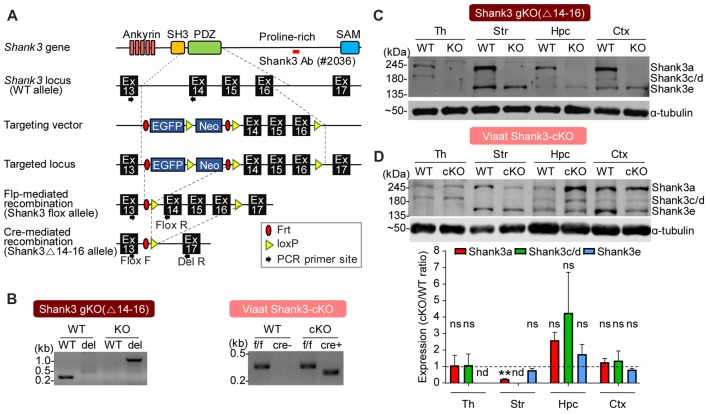
Generation and characterization of global *Shank3*^Δ14–16^ and *Viaat-Cre;Shank3*^Δ14–16^ mice. **(A)** conditional knockout (cKO) strategy for exons 14–16 of the *Shank3* gene in mice. Ankyrin, ankyrin repeats; SH3, src homology 3 domain; PDZ, PSD-95, Dlg, ZO-1 domain; Pro-rich, proline-rich region; SAM, sterile alpha motif; Ex, exon; Frt, flippase recombinase target; loxP, locus of X-over P1. **(B)** Polymerase chain reaction (PCR) genotyping of global *Shank3*^Δ14–16^ and *Viaat-Cre;Shank3*^Δ14–16^ mice. Note that the primer set targeting exons 13 and 14 generates a PCR band (276 bp) in the wild-type (WT) allele, but not in the *Shank3*^Δ14–16^ (*del*) allele, whereas the primer set targeting exons 13 and 17 generates a PCR band (1159 bp) in the *Shank3*^Δ14–16^ (*del*) allele, but not in the WT allele. **(C)** Immunoblot analyses of Shank3 protein splice variants in WT and *Shank3*^Δ14–16^ mice (13 weeks; male) using a Shank3-specific antibody (#2036); the target region is indicated in panel **A** as a red bar. Th, thalamus; Str, striatum; Hpc, hippocampus; Ctx, cortex. **(D)** Reduced levels of Shank3 protein variants in different brain regions of *Viaat-Cre;Shank3*^Δ14–16^ mice (12 weeks; female). Total brain lysates were analyzed by immunoblotting using a Shank3-specific antibody (#2036). cKO band signals normalized to α-tubulin were normalized to those from WT mice. Data are shown as mean ± SEM. *n* = 3 pairs (WT, cKO), **P < 0.01, nd, not detectable, ns, not significant and one sample *t*-test.

To analyze the effects of cell type-specific *Shank3* deletion, we first generated a new mouse line harboring a cassette containing exons 14–16 of the *Shank3* gene flanked by flox sequences, and then crossed these mice with *protamine-Flp and* *protamine-Cre* mice to produce mice in which *Shank3* exons 14–16 were globally and homozygously deleted (*Shank3*^Δ14–16^ mice; Figure 2A). PCR confirmed the genotype of these mice (Figure 2B), and immunoblot analyses revealed that the two main splice variants of the Shank3 protein (Shank3a and Shank3c/d) were absent in several brain regions (Figure 2C), a result expected based on previous studies on the alternative splicing of *Shank3* (Lim et al., 1999; Maunakea et al., 2010; Waga et al., 2014; Wang et al., 2014).

We next generated mice carrying *Shank3*^Δ14–16^ deletion restricted to GABAergic neurons by crossing *Shank3*^fl/fl^ mice with *Viaat-Cre* mouse lines, which drives gene expression globally in GABAergic neurons by the solute carrier family 32 (GABA vesicular transporter) member 1 (Slc32a1 or Viaat/vesicular inhibitory amino acid transporter) promoter (Chao et al., 2010; Kim et al., 2018). *Viaat-Cre;Shank3*^Δ14–16^ mice, genotyped by PCR (Figure 2B), showed a strong reduction in Shank3a in the striatum (Figure 2D), a brain region enriched with GABAergic neurons. Notably, the hippocampus displayed a strong tendency for an increase in *Shank3* expression, likely reflecting compensatory changes in the mutant pyramidal neurons caused by the *Shank3* deletion in GABAergic neurons in the hippocampus or other brain regions.

### Suppressed Excitatory Synaptic Transmission in the Dorsolateral Striatum in Global *Shank3*^Δ14–16^ and *Viaat-Cre;Shank3*^Δ14–16^ Mice

**Figure 3:**
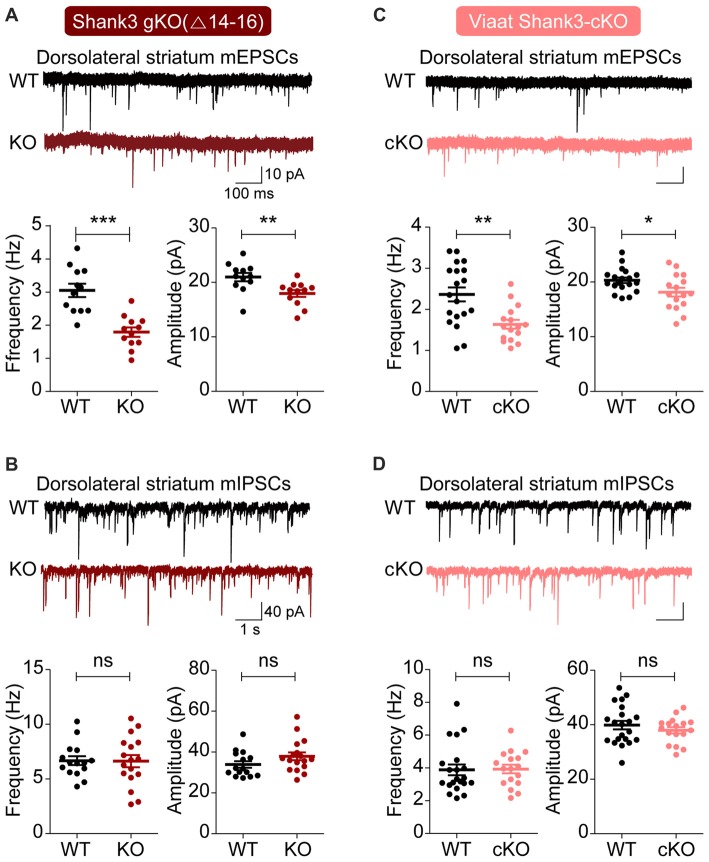
Suppressed excitatory but not inhibitory synaptic transmission in the dorsolateral striatum in global *Shank3*^Δ14–16^ and *Viaat-Cre;Shank3*^Δ14–16^mice. **(A,B)** Global *Shank3*^Δ14–16^ mice (P28–35) show decreased frequency and amplitude of mEPSCs, and normal mIPSCs in dorsolateral striatal neurons. Data are shown as mean ± SEM. *n* = 12 neurons from four mice (WT), 12, 3 (KO) for mEPSCs, 15, 4 (WT), 17, 4 (KO) for mIPSCs, **P < 0.01, ***P < 0.001, ns, not significant, Student’s *t*-test. **(C,D)** *Viaat-Cre;Shank3*^Δ14–16^ mice (P28–35) show decreased frequency and amplitude of mEPSCs, and normal mIPSCs in dorsolateral striatal neurons. *n* = 19, 5 (WT), 16, 4 (cKO) for mEPSCs, 21, 5 (WT), 17, 5 (cKO) for mIPSCs, *P < 0.05, **P < 0.01, ns, not significant, Student’s *t*-test (frequency and amplitude of mEPSCs, and amplitude of mIPSC), and Mann-Whitney U test (frequency of mIPSC).

We first measured excitatory and inhibitory synaptic transmission in the dorsal striatum, a region with enriched with GABAergic neurons and implicated in the development of abnormal behaviors in *Shank3*-mutant mice (Peca et al., 2011; Peixoto et al., 2016). Both the frequency and amplitude of mEPSCs were substantially decreased in dorsolateral striatal neurons in global *Shank3*^Δ14–16^ mice, with frequency exhibiting a larger decrease; in contrast, mIPSCs were normal (Figures 3A,B).

Similar changes were observed in dorsolateral striatal neurons in *Viaat-Cre;Shank3*^Δ14–16^ mice: mEPSC frequency and amplitude were decreased, whereas mIPSCs were normal (Figures 3C,D). Collectively, these results suggest that global and GABAergic *Shank3* deletions similarly suppress excitatory synaptic transmission in dorsolateral striatal neurons without affecting inhibitory synaptic transmission.

### Enhanced Direct Social Interaction and Suppressed Social Communication in Global *Shank3*^Δ14–16^ and *Viaat-Cre;Shank3*^Δ14–16^ Mice

**Figure 4:**
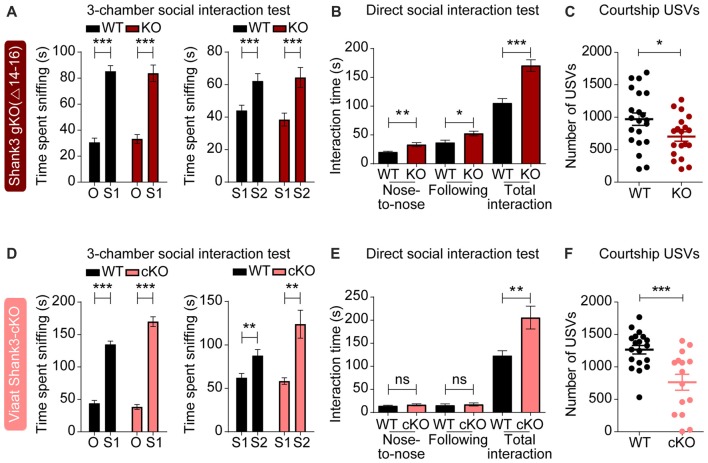
Enhanced direct social interaction and suppressed social communication in global *Shank3*^Δ14–16^ and *Viaat-Cre;Shank3*^Δ14–16^ mice. **(A)** Normal social approach and social novelty recognition in *Shank3*^Δ14–16^ mice (14–21 weeks) in the three-chamber test, as shown by time spent sniffing. S1, stranger; O, object; S2, novel stranger. Data are shown as mean ± SEM. *n* = 28 (WT) and 22 (KO), ***P < 0.001, paired *t*-test. Details on the order of behavioral tests performed on *Shank3*^Δ14–16^ mice and conditional *Shank3*^Δ14–16^ mouse lines (see below) are described in Supplementary Table S1. **(B)** Enhanced social interaction in *Shank3*^Δ14–16^ mice (14–20 weeks) in the direct social interaction test, as shown by nose-to-nose interaction, following and total interaction, the latter of which additionally includes allo-grooming and body contacts. Mean ± SEM. *n* = 20 (WT) and 16 (KO), *P < 0.05, **P < 0.01, ***P < 0.001, Student’s *t*-test. **(C)** Suppressed ultrasonic vocalizations (USVs) in *Shank3*^Δ14–16^ mice (18–24 weeks), upon encounter with a novel female stranger. n = 21 (WT) and 19 (KO), *P < 0.05, Student’s *t*-test. **(D–F)** *Viaat-Cre;Shank3*^Δ14–16^ mice (10–13 weeks for **D**, 15–28 weeks for **E**, and 11–21 weeks for **F**) show normal social approach in the three-chamber test **(D)** enhanced direct social interaction **(E)** and suppressed courtship USVs **(F)**. *n* = 19 mice (WT), 13 (cKO) for three-chamber, 11 (WT), 8 (cKO) for direct social interaction, and 19 (WT), 15 (cKO) for USV, **P < 0.01, ***P < 0.001, ns, not significant, paired *t*-test (three-chamber test), Mann-Whitney U test (nose to nose time and following time of direct social interaction test), and Student’s *t*-test (total interaction time of direct social interaction test and adult USV test).

To test the impact of global and GABA neuron-specific deletions of *Shank3* exons 14–16 on behaviors in mice, we subjected *Shank3*^Δ14–16^ and *Viaat-Cre;Shank3*^Δ14–16^ mice to a battery of behavioral tests. *Shank3*^Δ14–16^ mice displayed normal social approach behavior in the three-chamber test, but increased social interaction in the direct social interaction test (Figures 4A,B). These mice also showed suppressed USVs upon encounter with a novel female stranger (courtship USVs; Figure 4C).

*Viaat-Cre;Shank3*^Δ14–16^ mice showed normal social approach behavior in the three-chamber test but enhanced direct social interaction and suppressed courtship USVs (Figures 4D–F), similar to the behaviors observed in global *Shank3*^Δ14–16^ mice. These results suggest that social interaction phenotypes induced by global *Shank3*^Δ14–16^ deletion is largely recapitulated in mice with *Shank3* exons 14–16 deletion restricted to GABAergic neurons.

### Strongly Altered Repetitive Behaviors in Global *Shank3*^Δ14–16^ Are Partially Mimicked by *Viaat-Cre;Shank3*^Δ14–16^ Mice

**Figure 5:**
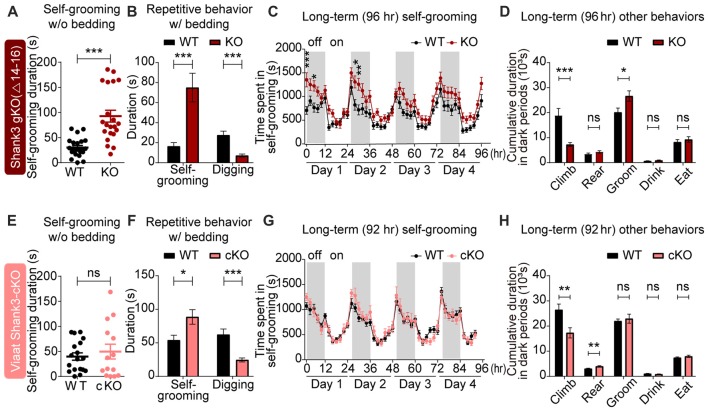
Strongly altered repetitive behaviors in global *Shank3*^Δ14–16^ mice are partially mimicked by *Viaat-Cre;Shank3*^Δ14–16^ mice. **(A)** Enhanced self-grooming in *Shank3*^Δ14–16^ mice (11–14 weeks) in a novel cage without bedding. *n* = 22 (WT) and 21 (KO), ***P < 0.001, Student’s *t*-test. **(B)** Enhanced self-grooming and suppressed digging in *Shank3*^Δ14–16^ mice (12–14 weeks) in home cages with bedding. *n* = 21 (WT) and 20 (KO), ***P < 0.001, Mann-Whitney U test. **(C)** Enhanced self-grooming in *Shank3*^Δ14–16^ mice (9–14 weeks) in Laboras cages, in which mouse movements are monitored for 4 days. *n* = 20 (WT) and 21 (KO), *P < 0.05, **P < 0.01, ***P < 0.001, repeated measures two-way ANOVA (genotype *p* value = 0.0149 and genotype × time *p* value < 0.0001). **(D)** Enhanced self-grooming and suppressed climbing in *Shank3*^Δ14–16^ mice (9–14 weeks) in Laboras cages (long-term monitoring). These graphs are a summary of all types of movements in Laboras cages for 4 days, including self-grooming. *n* = 20 (WT) and 21 (KO), *P < 0.05, ***P < 0.001, ns, not significant, Student’s *t*-test. **(E–H)** *Viaat-Cre;Shank3*^Δ14–16^ mice (11–15 weeks for **E**, 10–26 weeks (cohort 3: 10–20 weeks, cohort 4: 23–26 weeks) for **F**, 12–14 weeks for **G,H**) show enhanced self-grooming and suppressed digging in home cages with bedding **(F)** but normal self-grooming in a novel cage without bedding **(E)** and normal self-grooming but enhanced rearing and suppressed climbing in Laboras cages (long-term monitoring; **G,H**). *n* = 18 mice (WT), 14 (cKO) for w/o bedding, 14 (WT), 18 (cKO) for w/bedding, and 15 (WT), 13 (cKO) for Laboras, *P < 0.05, **P < 0.01, ***P < 0.001, repeated measures two-way ANOVA (for Laboras; genotype *p* value = 0.79), Student’s *t*-test (for self-grooming test, repetitive behavior test, and Laboras (climbing, rearing, grooming and drinking)), and Mann-Whitney U test (for Laboras (eating)).

In tests for repetitive behaviors, global *Shank3*^Δ14–16^ mice displayed enhanced self-grooming in a test cage without bedding (Figure 5A), and exhibited enhanced self-grooming but reduced digging in home cages with bedding (Figure 5B). In Laboras cages, where mouse movements are continuously monitored for 4 days, global *Shank3*^Δ14–16^ mice showed enhanced self-grooming, although climbing behavior was suppressed (Figures 5C,D).

*Viaat-Cre;Shank3*^Δ14–16^ mice showed enhanced self-grooming and suppressed digging in home cages with bedding and enhanced rearing in Laboras cages (long-term monitoring), but normal self-grooming in a novel cage without bedding as well as in Laboras cages (Figures 5E–H); these results differed in some respects from those of the global *Shank3*^Δ14–16^ mice.

Home-cage digging and Laboras-cage climbing were similarly reduced in global *Shank3*^Δ14–16^ and *Viaat-Cre;Shank3*^Δ14–16^ mice. These results suggest that the strong self-grooming induced by global *Shank3* deletion is not fully recapitulated by the GABAergic *Shank3* deletion, while the digging and climbing are similarly suppressed by both deletions.

### Similar Novelty-Induced Hypoactivity in Global *Shank3*^Δ14–16^ and *Viaat-Cre;Shank3*^Δ14–16^ Mice

**Figure 6:**
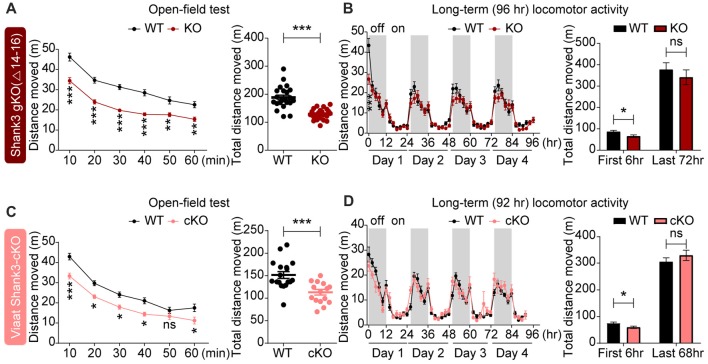
Novelty-induced hypoactivity in global *Shank3*^Δ14–16^ and *Viaat-Cre;Shank3*^Δ14–16^ mice. **(A)** Suppressed locomotor activity, as measured by distance moved in *Shank3*^Δ14–16^ mice (11–13 weeks) in the open-field test. Data are shown as mean ± SEM. *n* = 23 (WT) and 22 (KO), **P < 0.01, ***P < 0.001, repeated measures two-way ANOVA (genotype *p* value < 0.0001; genotype × time interaction *p* value = 0.0109) and Student’s *t*-test. **(B)** *Shank3*^Δ14–16^ mice (9–14 weeks) show suppressed locomotor activity during an early period (first 2 and 6 h), but not in the later habituated period (last 72 h), in Laboras cages (long-term monitoring). *n* = 20 (WT) and 21 (KO), *P < 0.05, ***P < 0.001, repeated measures two-way ANOVA (genotype *p* value = 0.3711; genotype × time interaction *p* value < 0.0001) and Student’s *t*-test. **(C,D)** *Viaat-Cre;Shank3*^Δ14–16^ mice (8–9 weeks for **C** and 12–14 weeks for **D**) show hypoactivity in the open-field test **(C)** and during the first 6 h but not the last 68 h in Laboras cages (long-term monitoring; **D**). *n* = 18 (WT), 15 (cKO) for open-field, 15 (WT), 13 (cKO) for Laboras, *P < 0.05, ***P < 0.001, ns, not significant, repeated measures two-way ANOVA (for the left panels in open-field and Laboras results; genotype *p* values = 0.0006 and 0.7414, time × genotype *p* values = 0.0454 and 0.0002, respectively), Student’s *t*-test.

In tests for locomotion, global *Shank3*^Δ14–16^ mice displayed reduced locomotor activity in the open-field test, a novel environment (Figure 6A). In Laboras cages (long-term monitoring), global *Shank3*^Δ14–16^ mice showed strong hypoactivity during the first 2 h and modest hypoactivity measured over the first 6 h; during the last 72 h, a period after full habitation to the environment, *Shank3*^Δ14–16^ mice exhibited normal locomotor activity (Figure 6B). These results suggest that *Shank3*^Δ14–16^ mice show hypoactivity in a novel, but not a familiar, environment.

*Viaat-Cre;Shank3*^Δ14–16^ mice showed decreased locomotor activity in the open-field test and during the first 6 h in Laboras cages (long-term monitoring; Figures 6C,D), similar to the novelty-induced hypoactivity in global *Shank3*^Δ14–16^ mice. These results suggest that global and GABAergic *Shank3* deletion lead to similar novelty-induced hypoactivity in mice.

### Partially Similar Anxiety-Like Behaviors in Global *Shank3*^Δ14–16^ and *Viaat-Cre;Shank3*^Δ14–16^ Mice

**Figure 7:**
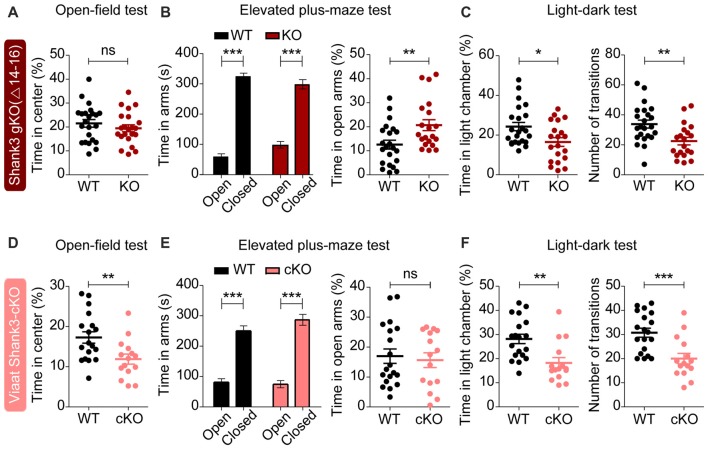
Partially similar anxiety-like behaviors in global *Shank3*^Δ14–16^ and *Viaat-Cre;Shank3*^Δ14–16^ mice. **(A)** Normal anxiety-like behavior in *Shank3*^Δ14–16^ mice (11–13 weeks), as measured by the time spent in the center region of the open field arena in the open-field test. Note that locomotor activity results from the open-field test are described in Figure 6A. Data are shown as mean ± SEM. *n* = 23 (WT) and 22 (KO), ns, not significant, Student’s *t*-test. **(B)** Anxiolytic-like behavior in *Shank3*^Δ14–16^ mice (13–21 weeks) in the elevated plus-maze test. *n* = 22 (WT) and 20 (KO), **P < 0.01, ***P < 0.001, paired *t*-test (for the left panels), and Student’s *t*-test (for the right panel). **(C)** Anxiety-like behavior in *Shank3*^Δ14–16^ mice (14–21 weeks) in the light-dark test. Note that these mice are also hypoactive in this test, as shown by the number of transitions. *n* = 23 (WT) and 20 (KO), *P < 0.05, **P < 0.01, Student’s *t*-test. **(D–F)** *Viaat-Cre;Shank3*^Δ14–16^ mice (8–9 weeks for **D**, 8–10 weeks for **E**, and 11–20 weeks for **F**) spend a reduced amount of time in the center region of the open-field area (**D**; locomotor activity results are described in Figure 6C), a normal amount of time in the open arm of the elevated plus-maze **(E)** and a decreased amount of time in the light chamber of the light-dark apparatus (**F**, left) and show decreased number of transitions (**F**, right). *n* = 18 (WT), 15 (cKO) for open-field, 19 (WT), 15 (cKO) for elevated plus-maze, and 19 (WT), 15 (cKO) for light-dark, **P < 0.01, ***P < 0.001, Student’s *t*-test (for open-field test, right panel of elevated-plus maze test, and number of transition of light-dark test), paired *t*-test (for the left panel of elevated plus-maze test), and Mann-Whitney U test (for time in light chamber of light-dark test).

In tests for anxiety-related behaviors, global *Shank3*^Δ14–16^ mice did not show anxiety-like behavior in the open-field test, as shown by the normal amount of time spent in the center region of the open-field arena (Figure 7A). However, these mice were less anxious in the elevated plus-maze test, spending more time in the open arm (Figure 7B), and, conversely, more anxious in the light-dark apparatus, spending less time in the light chamber (Figure 7C). *Shank3*^Δ14–16^ mice also showed a reduced number of transitions between light and dark chambers in the light-dark apparatus, in line with the hypoactivity of the mice. These results suggest that global *Shank3*^Δ14–16^ mice show differential anxiety-like behaviors.

*Viaat-Cre;Shank3*^Δ14–16^ mice were more anxious in open-field and light-dark tests, spending less amount of time in the center region of the open-field arena (Figure 7D) and in the light chamber of the light-dark apparatus (Figure 7F). However, these mice did not show anxiety-like behavior in the elevated plus-maze test, spending normal amount of time in the open arm (Figure 7E).

These results suggest that global and GABAergic *Shank3* deletions similarly induce anxiety-like behaviors in the light-dark test, whereas they have differential influences on other types of anxiety-like behaviors. Therefore, GABAergic *Shank3* deletion seems to strongly contribute to the anxiety-like behavior of global *Shank3*^Δ14–16^ mice in the light-dark test. These results also suggest that GABAergic *Shank3* deletion does induce anxiety-like behavior in the open-field test, but this is masked by global *Shank3* deletion. In contrast, GABAergic *Shank3* deletion seems to have minimal impacts on the anxiety-like behavior in the elevated plus-maze test, suggesting that non-GABAergic *Shank3* deletions are more important for the anxiolytic-like behaivor of global *Shank3*^Δ14–16^ mice in the elevated plus-maze.

## Discussion

In this study, we investigated the impacts of global and GABA neuron-specific deletion of *Shank3* exons 14–16 on synaptic transmission and behaviors in mice. Global *Shank3*^Δ14–16^ mice display decreased excitatory input onto dorsolateral striatal neurons and strong abnormalities in social, repetitive, locomotor and anxiety-like behaviors. The electrophysiological and behavioral (social and locomotor) phenotypes observed in global *Shank3*^Δ14–16^ mice are strongly mimicked by *Viaat-Cre;Shank3*^Δ14–16^ mice, although the repetitive and anxiety-like behavioral deficits in global *Shank3*^Δ14–16^ mice are partially mimicked by *Viaat-Cre;Shank3*^Δ14–16^ mice (summarized in Table 1).

**Table 1:** Summary of electrophysiological and behavioral phenotypes of global *Shank3*^Δ14–16^ and *Viaat-Cre;Shank3*^Δ14–16^ mice.

			*Shank3* exons 14-16 global KO		*Shank3* exons 14-16 Viaat cKO	
**Electrophysiology**	**Brain region**	**Measurement**	Frequency	Amplitude	Frequency	Amplitude
	Dorsolateral striatum	mEPSC	↓	↓	↓	↓
		mIPSC	-	-	-	-
**Behavior**	**Behavioral domain**	**Behavioral test**	***Shank3* exons 14-16 global KO**		***Shank3* exons 14-16 Viaat cKO**	
	Social interaction	3-chamber	-		-	
		Direct	Total interaction ↑		Total interaction ↑	
		interaction	Nose-to-nose ↑		Nose-to-nose, -	
			Following ↑		Following, -	
	Social communication	Adult USV (courtship)	↓		↓	
	Repetitive behavior	Laboras	Self-grooming ↑		Rearing ↑	
			Climbing ↓		Climbing ↓	
		Self-grooming (w/o bedding)	↑		-	
		Repetitive behavior	Self-grooming ↑		Self-grooming ↑	
		(with bedding)	Digging ↓		Digging ↓	
	Locomotor activity	Laboras (first 6 h)	↓		↓	
		Open-field	↓		↓	
	Anxiety-like behavior	Open field (center time)	-		↓	
		Elevated plus-maze (time in open arms)	↑		-	
		Light/dark box (time in light chamber)	↓		↓	

*This table summarizes only the increases or decreases of various electrophysiological and behavioral phenotypes in a given mouse line relative to wild-type (WT)/control mice, but is not intended to compare the phenotypic severities across different mouse lines. -, no significant change; up and down arrows, increases and decreases*.

The result that both the frequency and amplitude of mEPSCs are reduced in global *Shank3*^Δ14–16^ mice (Figure 3) further strengthens the notion that Shank3 is important for the development and function of excitatory synapses in the dorsal striatum. Similar decreases in the frequency and amplitude of mEPSCs in the dorsal striatum have been observed in the *Shank3* mouse line lacking exons 13–16 (*Shank3B*^−/−^ mice; Peca et al., 2011; Mei et al., 2016; Wang et al., 2017).

A more important finding from our study is that both mouse lines (global and *Viaat-Cre*) show similar decreases in the frequency and amplitude of mEPSCs in dorsolateral striatal neurons (Figure 3). This suggests that the suppressed excitatory input onto dorsolateral striatal neurons in these mouse lines are likely to be induced by the deletion of *Shank3* in striatal GABAergic neurons in a cell autonomous manner.

In support of this possibility, our FISH data indicate that *Shank3* mRNAs are expressed in Gad1/2-positive neurons in the dorsal striatum in addition to mPFC (Figure 1). In addition, the immunostaining result indicates that Shank3-positive punctate structures are observed on the dendrites of GAD67-positive GABAergic neurons in cultured hippocampal neurons. Given that Shank3 is an important component of the postsynaptic density at excitatory synapses (Sheng and Kim, 2000; Sheng and Sala, 2001; Boeckers et al., 2002; Sheng and Hoogenraad, 2007; Grabrucker et al., 2011; Sheng and Kim, 2011; Jiang and Ehlers, 2013; Sala et al., 2015; Monteiro and Feng, 2017; Mossa et al., 2017), the lack of Shank3 in dorsolateral striatal neurons may suppress normal development and maturation of the postsynaptic density, dendritic spines, and excitatory synapses. In addition, previous studies have reported a strong decrease in dendritic spine density in dorsal striatal neurons in *Shank3B*^−/−^ mice (Peca et al., 2011), further suggesting that the decreased mEPSC frequency may be a consequence of postsynaptic changes.

More recently, however, additional analyses of excitatory synaptic inputs onto D1 and D2 medium spiny neurons (MSNs) in the dorsal striatum of *Shank3B*^−/−^ mice have revealed that D2 MSNs show reductions in both presynaptic release and spine density (Wang et al., 2017), suggesting that both pre- and postsynaptic factors may be involved. In addition, a previous study on *Shank3B*^−/−^ mice showed that early abnormal excitability in pyramidal neurons in the somatosensory cortex driven by the limited inhibitory input from neighboring GABAergic neurons induces precocious development of excitatory synapses on dorsomedial striatal neurons that leads to a decrease in the mEPSC frequency at later stages (Peixoto et al., 2016). It is therefore possible that the decreased mEPSC frequency in dorsolateral striatal neurons in global *Shank3*^Δ14–16^ and *Viaat-Cre;Shank3*^Δ14–16^ mice may represent the consequences of the primary changes occurring in cortical GABAergic neurons.

Behaviorally, global *Shank3*^Δ14–16^ mice display altered social and repetitive behaviors, including suppressed courtship USVs and enhanced self-grooming (Figures 4, 5). These mice also show hypoactivity and altered anxiety-like behaviors (Figures 6, 7). Given that *Shank3*^Δ14–16^ mice lack the PDZ domain-containing Shank3 variants, Shank3a and Shank3c/d, but retain Shank3e, our mouse line is likely to display behavioral phenotypes similar to those observed in the *Shank3B*^−/−^ mouse line, which globally lacks exons 13–16 encoding the PDZ domain (Peca et al., 2011). Indeed, *Shank3*^Δ14–16^ and *Shank3B*^−/−^ mice show largely similar behaviors, including suppressed courtship USVs, hypoactivity, and anxiety-like behavior (elevated zero maze and light-dark test), although *Shank3B*^−/−^ mice additionally show suppressed social approach (Peca et al., 2011; Dhamne et al., 2017). Another *Shank3*-mutant mice similar to ours is the one lacking exon 13, encoding the PDZ domain (*Shank3*^E13^ mice; Jaramillo et al., 2017). These mice show enhanced self-grooming and social interaction deficits, but normal locomotion and anxiety-related behavior; the partial similarity to our behavioral phenotypes is likely attributable to the different exon targeting strategy (insertion of a stop codon in front of exon 13) in *Shank3*^E13^ mice.

Although the behavioral phenotypes of global *Shank3*^Δ14–16^ mice are strong in multiple domains (social, repetitive, locomotor, and anxiety-like), the following points need to be further discussed. First, global *Shank3*^Δ14–16^ mice show enhanced direct social interaction, which was unexpected and is at variance with the normal three-chamber social approach observed in these mice. Notably, a previous study on *Shank3*^Δ4–22^ mice has reported a similar increase in direct social interaction where *Shank3*^Δ4–22^ mice display frequently attempted but unsuccessful social interactions with a stranger C3H mouse, a different strain, that does not reciprocate and terminate the social interaction attempted by the subject mouse (Wang et al., 2016), suggesting that *Shank3*^Δ4–22^ mice have normal social interest but struggle with persisting social failures. We could not test whether this is the case for our mice because we used genotype-matched (WT-WT or KO-KO) mouse pairs where monitoring of non-reciprocated social interaction is difficult because of the same coat color and the confusion over retraction vs. rejection. However, our results suggest that social interest is normal in global *Shank3*^Δ14–16^ mice, which is different from the significant social interaction deficits observed in many other *Shank3*-mutant mouse lines (Jiang and Ehlers, 2013; Monteiro and Feng, 2017). We propose that the difference in the specific *Shank3* exons deleted in each mouse lines might explain the discrepancy. For instance, the exons deleted in our *Shank3* mice (exons 14–16) are distinct from those deleted in *Shank3B*^−/−^ mice (exon 13–16; Peca et al., 2011). In support of this possibility, a very small difference in the exons deleted in *Shank2*-mutant mice (i.e., exons 6 and 7 vs. exon 7) has been shown to cause strong differences in molecular, synaptic and behavioral phenotypes (Schmeisser et al., 2012; Won et al., 2012; Lim et al., 2017; Wegener et al., 2018).

Another notable result is that global *Shank3*^Δ14–16^ mice display anxiolytic-like behavior in the elevated plus-maze whereas they show anxiety-like behavior in the light-dark apparatus and normal anxiety-like behavior in the center region of open-field arena. This could be due to the different anxiogenic components in these tests (Belzung and Griebel, 2001; Carola et al., 2002; Carobrez and Bertoglio, 2005), as exemplified by the differential responses of nine different mouse strains to the elevated plus-maze and light-dark tests (Griebel et al., 2000). Notably, *Shank3B*^−/−^ mice (exons 13–16) also display differential anxiety-like behaviors in these assays, being partly similar to our results; normal anxiety-like behavior in elevated plus-maze, anxiety-like behavior in zero maze, light-dark apparatus and open-field center (Peca et al., 2011; Dhamne et al., 2017).

Our results indicate that GABAergic neurons contribute to some of the abnormal behaviors observed in global *Shank3*^Δ14–16^ mice. Specifically, the enhanced direct social interaction, suppressed courtship USVs, and novelty-induced hypoactivity observed in global *Shank3*^Δ14–16^ mice were also observed in *Viaat-Cre;Shank3*^Δ14–16^ mice. In contrast, the strong self-grooming behavior observed in global *Shank3*^Δ14–16^ mice were only partially mimicked by *Viaat-Cre;Shank3*^Δ14–16^ mice. In anxiety-like behaviors, only the light-dark test results were similar in global *Shank3*^Δ14–16^ and *Viaat-Cre;Shank3*^Δ14–16^ mice. Therefore, GABA neuronal *Shank3* deletion seems to be more important for social and locomotor behaviors than repetitive and anxiety-like behaviors.

A recent study reported the effects of a deletion of *Shank3* exons 4–22 restricted to Nex-positive glutamatergic neurons in the cortex, hippocampus and amygdala (*Nex-Shank3* cKO mice) and Dlx5/6-positive GABAergic neurons in the striatum (*Dlx5/6-Shank3* cKO mice; Bey et al., 2018). Neither *Nex-Shank3* nor *Dlx5/6-Shank3* cKO mice exhibit social approach deficits, results similar to the normal social approach behavior reported by the same group using mice with a global *Shank3*^Δ4–22^ mice (Wang et al., 2016). This phenotype is also similar to the normal social approach behavior observed in our global *Shank3*^Δ14–16^ and *Viaat-Cre;Shank3*^Δ14–16^ mice.

The suppressed courtship USV and hypoactivity phenotypes observed in global *Shank3*^Δ4–22^ mice (Wang et al., 2016) were not recapitulated in either *Nex-Shank3* or *Dlx5/6-Shank3* cKO mice (Bey et al., 2018). These results are different from our findings that both global *Shank3*^Δ14–16^ and *Viaat-Cre;Shank3*^Δ14–16^ mice show suppressed courtship USV and hypoactivity. Furthermore, the enhanced self-grooming observed in global *Shank3*^Δ4–22^ mice (Wang et al., 2016) was observed in *Nex-Shank3* cKO mice, but not in *Dlx5/6-Shank3* cKO mice (Bey et al., 2018). These results are slightly different from our finding that the enhanced self-grooming in global *Shank3*^Δ14–16^ mice was partially recapitulated in *Viaat-Cre;Shank3*^Δ14–16^mice.

These results indicate that two different global *Shank3* deletions (exons 14–16 and 4–22) in mice lead to remarkably similar behavioral phenotypes in mice in social, repetitive, locomotor and anxiety-like behavioral domains, but that these similarities are minimized by two different cKOs restricted to GABAergic neurons (Dlx5/6 and Viaat). These discrepancies could be attributable to differences in the specific exons of *Shank3* deleted and/or specific characteristics of *Dlx5/6-Cre* vs. *Viaat-Cre* mice (Oh et al., 2005; Goebbels et al., 2006; Chao et al., 2010). For instance, *Dlx5/6-Cre* primarily targets GABAergic neurons in the striatum (Monory et al., 2006), whereas *Viaat-Cre* targets the majority of GABAergic neurons in the brain (Chao et al., 2010). In addition, it could be subtle differences in mouse housing conditions or experimental details.

It remains unclear how GABA neuronal deletion of *Shank3* (exons 14–16) leads to the above mentioned diverse behavioral abnormalities. However, functional defects in the striatum have been strongly implicated in abnormal phenotypes in various *Shank3*-mutant mouse lines (Peca et al., 2011; Schmeisser et al., 2012; Filice et al., 2016; Jaramillo et al., 2016; Mei et al., 2016; Peixoto et al., 2016; Sarowar et al., 2016; Zhou et al., 2016; Jaramillo et al., 2017; Lee Y. et al., 2017; Reim et al., 2017; Vicidomini et al., 2017; Wang et al., 2017; Bey et al., 2018). In addition, a recent study has shown that chemogenetic stimulation of D2, but not D1, MSN activity by DREADD-hM3Dq for the activation of the striatopallidal pathway can rescue self-grooming in *Shank3B*^−/−^ mice (Wang et al., 2017). Therefore, the suppressed excitatory synaptic transmission in dorsolateral striatal neurons in global *Shank3*^Δ14–16^ and *Viaat-Cre;Shank3*^Δ14–16^ mice might have contributed to the behavioral abnormalities observed in our mouse lines, including enhanced self-grooming. However, the substantial difference between the strong self-grooming in global *Shank3*^Δ14–16^ mice and the weak self-grooming in *Viaat-Cre;Shank3*^Δ14–16^ mice suggests that GABAergic *Shank3* deletion only partially contribute to the self-grooming phenotype. However, care should be taken in the interpretation because Viaat-mediated GABAergic *Shank3* deletion can affect multiple types of GABAergic neurons.

In conclusion, our results suggest that the deletion of *Shank3* exons 14–16 restricted to GABAergic neurons in mice induces phenotypes that are similar to those induced by global *Shank3* deletion. These include strongly suppressed excitatory synaptic onto dorsolateral striatal neurons and strongly altered social and locomotor behaviors but modestly altered repetitive and anxiety-like behaviors.

## Author Contributions

TY, JL and HC performed behavioral experiments. TY and JL performed immunoblot experiments. TY, HP and Y-EY performed electrophysiological experiments. EY and JK performed *in situ* hybridization experiments. TY performed hippocampal neuron culture and immunocytochemical experiments. HK and EK designed research and wrote the manuscript.

## Conflict of Interest Statement

The authors declare that the research was conducted in the absence of any commercial or financial relationships that could be construed as a potential conflict of interest. The reviewer RW declared a past co-authorship with one of the authors EK to the handling Editor.
