# Supplementary material for: GABA Neuronal Deletion of Shank3 Exons 14–16 in Mice Suppresses Striatal Excitatory Synaptic Input and Induces Social and Locomotor Abnormalities
Source: Front Cell Neurosci. 2018 Oct 9;12:341. doi: 10.3389/fncel.2018.00341 (PMC6189516; doi:10.3389/fncel.2018.00341)
Supplement: Supplementary file 1 [file Data_Sheet_1.PDF]

Supplementary Table 1. The order of behavior experiments

This table shows the detailed order of behavior experiments of each lines.

1. The order of behavior experiments for *Shank3*$^{\Delta 14\text{-}16}$									
Cohort1	Open-field test	Self-grooming	Repetitive behavior	Elevated plus-maze	Laboras test	Light-dark test	Direct interaction	Adult USV	
Cohort2	Laboras test	Open-field test	Self-grooming	Repetitive behavior	3-chamber test	Direct interaction	Elevated plus-maze	Light-dark test	Adult USV
Cohort3_1	3-chamber test	Direct interaction							
Cohort3_2	Direct interaction	3-chamber test							
2. The order of behavior experiments for *Viaat-Cre;Shank3*$^{\Delta 14\text{-}16}$									
Cohort1	Open-field test	Elevated plus-maze	3-chamber test	Adult USV	Self-grooming	Light-dark test	Direct interaction		
Cohort2	Open-field test	Elevated plus-maze	Self-grooming	Light-dark test	3-chamber test	Laboras test	Direct interaction	Adult USV	
Cohort3	Laboras test	Repetitive behavior							
Cohort4	Repetitive behavior								
Cohort5	Direct interaction								
