# Supplementary material for: GABA Neuronal Deletion of Shank3 Exons 14–16 in Mice Suppresses Striatal Excitatory Synaptic Input and Induces Social and Locomotor Abnormalities
Source: Front Cell Neurosci. 2018 Oct 9;12:341. doi: 10.3389/fncel.2018.00341 (PMC6189516; doi:10.3389/fncel.2018.00341)
Supplement: Supplementary file 2 [file Data_Sheet_2.PDF]

Supplementary Table 2. Statistics table

This table shows the details of statistical analyses and results

Figure		Assay Performed	Parameter (Unit)	Comparison					DescriptiveStatistics	Statistical Analysis					Normality test p values
				Variables 1	Variables 2	Age	n (animals)	Sex	Average ± SEM	Normality test	Statistical Test	Significance			
2	D	Western blot	Expression (cKO/WT ratio)	Th	Shank3 a	12 weeks	3 pairs	Female	1.057220 ± 0.6214010		One sample t-test (Theoretical mean : 1.00)	t(2) = 0.09208184	Two-tailed	P = 0.935; ns	
					Shank3 c/d				1.078044 ± 0.6792321			t(2) = 0.1149006	Two-tailed	P = 0.919; ns	
					Shank3 e				not detectable						
				Str	Shank3 a				0.2334660 ± 0.03662507			t(2) = 20.92922	Two-tailed	P = 0.0023; **	
					Shank3 c/d				not detectable						
					Shank3 e				0.7681456 ± 0.1092577			t(2) = 2.122089	Two-tailed	P = 0.1679; ns	
				Hpc	Shank3 a				2.587107 ± 0.4915103			t(2) = 3.229041	Two-tailed	P = 0.084; ns	
					Shank3 c/d				4.249126 ± 2.465153			t(2) = 1.318022	Two-tailed	P = 0.3182; ns	
					Shank3 e				1.744348 ± 0.5977580			t(2) = 1.245233	Two-tailed	P = 0.3392; ns	
				Ctx	Shank3 a				1.252051 ± 0.2415029			t(2) = 1.043676	Two-tailed	P = 0.4062; ns	
					Shank3 c/d				1.351464 ± 0.5862461			t(2) = 0.5995160	Two-tailed	P = 0.6097; ns	
					Shank3 e				0.8031158 ± 0.08167858			t(2) = 2.410475	Two-tailed	P = 0.1375; ns	
3	A	Dorsolateral striatum mEPSCs	Frequency (Hz)	WT		P28-35	WT = 12 (4)<br>KO = 12 (3)	Male	3.053472 ± 0.2016881	O	Student's t-test	t(22) = 5.109913	Two-tailed	P < 0.0001; ***	Yes; P = 0.7416; ns
				KO					1.791667 ± 0.1424702						Yes; P = 0.9513; ns
			Amplitude (pA)	WT					20.95888 ± 0.7680289	O	Student's t-test	t(22) = 3.006345	Two-tailed	P = 0.0065; **	Yes; P = 0.0804; ns
				KO					17.95469 ± 0.6392927						Yes; P = 0.3276; ns
	B	Dorsolateral striatum mIPSCs	Frequency (Hz)	WT		P28-35	WT = 15 (4)<br>KO = 17 (4)	Male	6.664444 ± 0.4062503	O	Student's t-test	t(30) = 0.0623372	Two-tailed	P = 0.9507; ns	Yes; P = 0.1919; ns
				KO					6.620588 ± 0.5546166						Yes; P = 0.8740; ns
			Amplitude (pA)	WT					33.84956 ± 1.607398	O	Student's t-test	t(30) = 1.592151	Two-tailed	P = 0.1218; ns	Yes; P = 0.1739; ns
				KO					37.88675 ± 1.912148						Yes; P = 0.0887; ns
	C	Dorsolateral striatum mEPSCs	Frequency (Hz)	WT		P28-35	WT = 19 (5)<br>cKO = 16 (4)	Male	2.362719 ± 0.1703913	O	Student's t-test	t(33) = 3.448454	Two-tailed	P = 0.0016; **	Yes; P = 0.3682; ns
				cKO					1.634896 ± 0.1087551						Yes; P = 0.2846; ns
			Amplitude (pA)	WT					20.28786 ± 0.4974168	O	Student's t-test	t(33) = 2.365769	Two-tailed	P = 0.0240; *	Yes; P = 0.4120; ns
				cKO					18.11959 ± 0.8066149						Yes; P = 0.8818; ns
	D	Dorsolateral striatum mIPSCs	Frequency (Hz)	WT		P28-35	WT = 21 (5)<br>cKO = 17 (5)	Male	3.883333 ± 0.3295369	X	Mann-Whitney U test	U = 153.0000	Two-tailed	P = 0.4630; ns	No; P = 0.0185; *
				cKO					3.926961 ± 0.2615632						Yes; P = 0.8913; ns
			Amplitude (pA)	WT					39.83214 ± 1.559193	O	Student's t-test	t(36) = 0.9381590	Two-tailed	P = 0.3544; ns	Yes; P = 0.7736; ns
				cKO					37.93575 ± 1.15187						Yes; P = 0.8030; ns
4	A	3-chamber social interaction test (O-S1)	Time spent sniffing (s)	WT	O	14-21 weeks	WT = 28<br>KO = 22	Male	30.59901 ± 3.236799	O	Paired t-test	t(27) = 10.48388	Two-tailed	P < 0.0001; ***	Yes; P = 0.4237; ns
					S1				85.2263 ± 4.428818						Yes; P = 0.2110; ns
				KO	O				33.25377 ± 3.380071	O	Paired t-test	t(27) = 3.886928	Two-tailed	P = 0.0006; ***	Yes; P = 0.6866; ns
					S1				83.68492 ± 6.368033						Yes; P = 0.6309; ns
		3-chamber social interaction test (S1-S2)	Time spent sniffing (s)	WT	S1				44.07887 ± 3.21724	O	Paired t-test	t(21) = 7.239439	Two-tailed	P < 0.0001; ***	Yes; P = 0.9639; ns
					S2				62.20614 ± 4.564713						Yes; P = 0.2237; ns
				KO	S1				38.4892 ± 3.974384	O	Paired t-test	t(21) =3.947851	Two-tailed	P = 0.0007; ***	Yes; P = 0.4636; ns
					S2				64.34786 ± 6.172367						Yes; P = 0.4933; ns
	B	Direct social interaction test	Interaction time (s)	Nose to nose	WT	14-20 weeks	WT = 20<br>KO = 16	Male	20.2655 ± 1.416918	O	Student's t-test	t(34) = 3.578579	Two-tailed	P = 0.0011; **	Yes; P = 0.9036; ns
					KO				33.04 ± 3.587167						Yes; P = 0.1026; ns
				Following	WT				36.6785 ± 4.18695	O	Student's t-test	t(34) = 2.679009	Two-tailed	P = 0.0113; *	Yes; P = 0.4134; ns
					KO				52.56062 ± 4.06208						Yes; P = 0.7344; ns
				Total interaction	WT				105.3495 ± 7.900419	O	Student's t-test	t(34) = 5.201175	Two-tailed	P < 0.0001; ***	Yes; P = 0.9539; ns
					KO				170.5494 ± 9.952661						Yes; P = 0.6266; ns
	C	Courtship USVs	Number of USVs	WT		18-24 weeks	WT = 21<br>KO = 19	Male	970.5714 ± 95.04499	O	Student's t-test	t(38) = 2.207	Two-tailed	P = 0.0334 ; *	Yes; P = 0.7514; ns
				KO					702.3684 ± 72.62494						Yes; P = 0.6309; ns
	D	3-chamber social interaction test (O-S1)	Time spent sniffing (s)	WT	O	10-13 weeks	WT = 19<br>cKO = 13	Male	43.93952 ± 4.579829	O	Paired t-test	t(18) = 10.41270	Two-tailed	P < 0.0001; ***	Yes; P = 0.8214; ns
					S1				134.7423 ± 5.087254						Yes; P = 0.1535; ns
				cKO	O				38.36064 ± 3.748086	O	Paired t-test	t(12) = 13.92911	Two-tailed	P < 0.0001; ***	Yes; P = 0.9073; ns
					S1				169.9381 ± 7.464016						Yes; P = 0.4551; ns
		3-chamber social interaction test (S1-S2)	Time spent sniffing (s)	WT	S1				62.09762 ± 5.088614	O	Paired t-test	t(18) = 2.938380	Two-tailed	P = 0.0088; **	Yes; P = 0.9508; ns
					S2				87.76311 ± 6.995053						Yes; P = 0.7727; ns
				cKO	S1				60.53106 ± 3.372473	O	Paired t-test	t(12) = 3.629994	Two-tailed	P = 0.0035; **	Yes; P = 0.4309; ns
					S2				115.9032 ± 14.9574						Yes; P = 0.7692; ns
	E	Direct social interaction test	Interaction time (s)	Nose to nose	WT	15-28 weeks	WT = 11<br>cKO = 8	Male	14.13636 ± 1.191156	X	Mann-Whitney U test	U = 33.00000	Two-tailed	P = 0.3859; ns	Yes; P = 0.7645; ns
					cKO				16.67375 ± 2.042861						No; P = 0.0391; *
				Following	WT				18.12545 ± 3.034415	X	Mann-Whitney U test	U = 38.00000	Two-tailed	P = 0.6497; ns	Yes; P = 0.1572; ns
					cKO				17.92375 ± 2.982247						No; P = 0.0209; *
				Total interaction	WT				112.3400 ± 11.35593	O	Student's t-test	t(17) = 3.048879	Two-tailed	P = 0.0073; **	Yes; P = 0.3954; ns
					cKO				190.9350 ± 26.09635						Yes; P = 0.8364; ns
	F	Courtship USVs	Number of USVs	WT		11-21 weeks	WT = 19<br>cKO = 15	Male	1264.474 ± 65.9612	O	Student's t-test	t(32) = 3.800742	Two-tailed	P = 0.0006; ***	Yes; P = 0.2151; ns
				cKO					763.6667 ± 122.8461						Yes; P = 0.2052; ns
	A	Self-grooming without bedding	Self-grooming duration (s)	WT		11-14 weeks	WT = 22<br>KO = 21	Male	30.27409 ± 4.003184	O	Student's t-test	t(41) = 5.139006	Two-tailed	P < 0.0001 ; ***	Yes; P = 0.5628; ns
				KO					92.62334 ± 11.69442						Yes; P = 0.1851; ns
	B	Repetitive behavior with bedding	Duration (s)	Self-grooming	WT	12-14 weeks	WT = 21<br>KO = 20	Male	16.43571 ± 3.658473	X	Mann-Whitney U test	U = 58.00000	Two-tailed	P < 0.0001 ; ***	Yes; P = 0.1930; ns
					KO				75.06 ± 14.27736						No; P = 0.0075; *
				Digging	WT				27.5381 ± 4.032623	X	Mann-Whitney U test	U = 43.00000	Two-tailed	P < 0.0001 ; ***	No; P = 0.0338; *
					KO				7.185 ± 1.509758						No; P = 0.0347; *
	C	Long-term (96 hr) self-grooming	Time spent in self-grooming (s)	2	WT	9-14 weeks	WT = 20<br>KO = 21	Male	706.9794		Repeated measures of two-way ANOVA; Bonferroni's multiple comparison test	Interaction<br>F(47, 1833) = 2.086581, P < 0.0001 ; ***<br><br>Genotype<br>F(1, 39) = 6.484575, P = 0.0149; *<br><br>Time<br>F(47, 1833) = 25.09093, P < 0.0001 ; ***		P < 0.001 ; ***	
					KO				1353.615						
				4	WT				909.2246					P > 0.05 ; ns	
					KO				1251.386						
				6	WT				767.5745					P < 0.05 ; *	
					KO				1224.578						
				8	WT				744.2985					P > 0.05 ; ns	
					KO				1105.438						
				10	WT				866.118					P > 0.05 ; ns	
					KO				968.6575						
				12	WT				967.6281					P > 0.05 ; ns	
					KO				1131.953						
				14	WT				346.951					P > 0.05 ; ns	
					KO				724.7562						
				16	WT				421.8865					P > 0.05 ; ns	
					KO				645.6376						
				18	WT				414.3065					P > 0.05 ; ns	
					KO				467.1857						
				20	WT				470.9505					P > 0.05 ; ns	
					KO				411.0995						
				22	WT				631.7374					P > 0.05 ; ns	
					KO				665.2618						
				24	WT				609.6185					P > 0.05 ; ns	
					KO				677.8101						
				26	WT				1193.276					P > 0.05 ; ns	
					KO				1499.894						
				28	WT				824.27					P < 0.05 ; *	
					KO				1309.678						
				30	WT				718.031					P < 0.01 ; **	
					KO				1259.96						
				32	WT				740.814					P > 0.05 ; ns	
					KO				1124.923						
				34	WT				668.0635					P > 0.05 ; ns	
					KO				895.1419						
				36	WT				635.9045					P > 0.05 ; ns	
					KO				1003.998						
				38	WT				382.3755					P > 0.05 ; ns	
					KO				573.6057						
				40	WT				407.213					P > 0.05 ; ns	
					KO				673.0438						
				42	WT				361.4815					P > 0.05 ; ns	
					KO				499.3071						
				44	WT				362.505					P > 0.05 ; ns	
					KO				548.4947						
				46	WT				579.226					P > 0.05 ; ns	
					KO				527.7719						
				48	WT				731.214					P > 0.05 ; ns	
					KO				662.7662						
				50	WT				994.995					P > 0.05 ; ns	
					KO				1058.177						
				52	WT				870.384					P > 0.05 ; ns	
					KO				1171.736						
				54	WT				662.566					P > 0.05 ; ns	
					KO				1053.692						
				56	WT				621.8649					P > 0.05 ; ns	
					KO				843.6548						
				58	WT				597.8654					P > 0.05 ; ns	
					KO				799.4919						
				60	WT				680.338					P > 0.05 ; ns	
					KO				918.8881						
				62	WT				369.887					P > 0.05 ; ns	
					KO				643.5219						
				64	WT				374.902					P > 0.05 ; ns	
					KO				519.1019						
				66	WT				350.531					P > 0.05 ; ns	
					KO				510.831						
				68	WT				441.2915					P > 0.05 ; ns	
					KO				508.3715						
				70	WT				713.8036					P > 0.05 ; ns	
					KO				908.1948						
				72	WT				868.551					P > 0.05 ; ns	
					KO				1166.73						
				74	WT				1152.649					P > 0.05 ; ns	
					KO				1391.513						
				76	WT				802.1125					P > 0.05 ; ns	
					KO				1107.824						
				78	WT				805.5475					P > 0.05 ; ns	
					KO				1083.876						
				80	WT				710.399					P > 0.05 ; ns	
					KO				1082.06						
				82	WT				835.875					P > 0.05 ; ns	
					KO				1043.355						
				84	WT				755.4244					P > 0.05 ; ns	
					KO				950.132						
				86	WT				279.5845					P > 0.05 ; ns	
					KO				559.9095						
				88	WT				296.5835					P > 0.05 ; ns	
					KO				512.7205						
				90	WT				338.226					P > 0.05 ; ns	
					KO				578.1428						
				92	WT				391.1925					P > 0.05 ; ns	

5				92	KO				545.021					P > 0.05 ; ns	
				94	WT				598.356					P > 0.05 ; ns	
					KO				830.5053						
				96	WT				910.5265					P > 0.05 ; ns	
					KO				1275.887						
	D	Long-term (96 hr) other behaviors	Cumulative duration in dark periods (10^3s)	Climbing	WT	9-14 weeks	WT = 20<br>KO = 21	Male	18877.18 ± 2816.685	O	Student's t-test	t(39) = 4.064162	Two-tailed	P = 0.0002; ***	Yes; P = 0.7600; ns
					KO				7254.35 ± 775.6096						Yes; P = 0.0953; ns
				Rearing	WT				3485.208 ± 372.7405	O	Student's t-test	t(39) = 1.365721	Two-tailed	P = 0.1799; ns	Yes; P = 0.1158; ns
					KO				4305.03 ± 465.8981						Yes; P = 0.7946; ns
				Grooming	WT				20165.78 ± 1687.115	O	Student's t-test	t(39) = 2.407094	Two-tailed	P = 0.0209; *	Yes; P = 0.5609; ns
					KO				26633.62 ± 2071.825						Yes; P = 0.8032; ns
				Driking	WT				676.2285 ± 109.9736	O	Student's t-test	t(39) = 0.9628907	Two-tailed	P = 0.3415; ns	Yes; P = 0.6742; ns
					KO				892.3143 ± 192.2562						Yes; P = 0.2130; ns
				Eating	WT				8242.735 ± 1011.358	O	Student's t-test	t(39) = 0.6975116	Two-tailed	P = 0.4896; ns	Yes; P = 0.5373; ns
					KO				9292.02 ± 1107.779						Yes; P = 0.6389; ns
	E	Self-grooming without bedding	Self-grooming duration (s)	WT		11-15 weeks	WT = 18<br>cKO = 14	Male	39.73167 ± 7.336972	O	Student's t-test	t(30) = 0.6416796	Two-tailed	P = 0.5260; ns	Yes; P = 0.1393; ns
				cKO					49.61071 ± 14.73665						Yes; P = 0.2570; ns
	F	Repetitive behavior with bedding	Duration (s)	Self-grooming	WT	10-26 weeks	WT = 14<br>cKO = 18	Male	54.04714 ± 7.416879	O	Student's t-test	t(30) = 2.605885	Two-tailed	P = 0.0141; *	Yes; P = 0.8406; ns
					cKO				91.26111 ± 11.16848						Yes; P = 0.8969; ns
				Digging	WT				62.26929 ± 8.437709	O	Student's t-test	t(30) = 4.549178	Two-tailed	P < 0.0001; ***	Yes; P = 0.6603; ns
					cKO				25.06278 ± 3.049526						Yes; P = 0.1592; ns
	G	Long-term (92 hr) self-grooming	Time spent in self-grooming (s)	2	WT	12-14 weeks	WT = 15<br>cKO = 13	Male	1072.813		Repeated measures of two-way ANOVA	Interaction<br>F(45, 1170) = 1.225201, P = 0.149; ns<br><br>Genotype<br>F(1, 26) = 0.07238321, P = 0.79; ns<br><br>Time<br>F(45, 1170) = 31.98375, P < 0.0001; ***			
					cKO				1249.342						
				4	WT				996.222						
					cKO				1142.316						
				6	WT				1023.263						
					cKO				951.4086						
				8	WT				814.7787						
					cKO				825.2569						
				10	WT				670.7206						
					cKO				590.4969						
				12	WT				875.1						
					cKO				725.347						
				14	WT				556.538						
					cKO				588.8962						
				16	WT				370.39						
					cKO				391.4053						
				18	WT				400.9113						
					cKO				360.8623						
				20	WT				452.1674						
					cKO				400.9723						
				22	WT				565.1854						
					cKO				614.3077						
				24	WT				688.66						
					cKO				721.8217						
				26	WT				1121.319						
					cKO				1329.031						
				28	WT				1034.214						
					cKO				1278.808						
				30	WT				832.4453						
					cKO				1012.653						
				32	WT				829.8934						
					cKO				858.9291						
				34	WT				734.32						
					cKO				829.4669						
				36	WT				742.6941						
					cKO				948.1729						
				38	WT				424.2047						
					cKO				570.6801						
				40	WT				326.4687						
					cKO				388.0815						
				42	WT				377.2554						
					cKO				326.2015						
				44	WT				423.3406						
					cKO				537.9607						
				46	WT				536.3647						
					cKO				575.9847						
				48	WT				629.6373						
					cKO				653.2115						
				50	WT				1160.103						
					cKO				1283.039						
				52	WT				991.6281						
					cKO				1065.906						
				54	WT				768.1279						
					cKO				818.877						
				56	WT				844.1434						
					cKO				827.6393						
				58	WT				691.4866						
					cKO				771.8054						
				60	WT				806.9647						
					cKO				769.8423						
				62	WT				519.2607						
					cKO				436.1854						
				64	WT				347.392						
					cKO				365.3984						
				66	WT				446.5493						
					cKO				374.1654						
				68	WT				591.8366						
					cKO				360.6146						
				70	WT				603.7187						
					cKO				439.9216						
				72	WT				568.6253						
					cKO				675.6592						
				74	WT				1362.294						
					cKO				1238.517						
				76	WT				1007.59						
					cKO				977.1546						
				78	WT				885.62						
					cKO				870.5862						
				80	WT				862.374						
					cKO				893.9548						
				82	WT				895.4841						
					cKO				789.6361						
				84	WT				972.7847						
					cKO				893.7955						
				86	WT				478.9613						
					cKO				459.7023						
				88	WT				339.694						
					cKO				395.5939						
				90	WT				471.3473						
					cKO				336.2446						
				92	WT				533.2567						
					cKO				494.5692						
	H	Long-term (92 hr) other behaviors	Cumulative duration in dark period (103s)	Climbing	WT	12-14 weeks	WT = 15<br>cKO = 13	Male	26472.56 ± 2288.466	O	Student's t-test	t(26) = 2.955590	Two-tailed	P = 0.0066; **	Yes; P = 0.4073; ns
					cKO				17302.49 ± 2030.616						Yes; P = 0.2440; ns
				Rearing	WT				3055.451 ± 168.6425	O	Student's t-test	t(26) = 3.179746	Two-tailed	P = 0.0038; **	Yes; P = 0.7842; ns
					cKO				3969.305 ± 239.974						Yes; P = 0.5346; ns
				Grooming	WT				21996.38 ± 820.8754	O	Student's t-test	t(26) = 0.4963072	Two-tailed	P = 0.6238; ns	Yes; P = 0.5023; ns
					cKO				22941.98 ± 1818.338						Yes; P = 0.5881; ns
				Drinking	WT				1043.731 ± 97.98065	O	Student's t-test	t(26) = 1.339522	Two-tailed	P = 0.1920; ns	Yes; P = 0.2868; ns
					cKO				841.0707 ± 116.8118						Yes; P = 0.6516; ns
				Eating	WT				7429.708 ± 299.7263	X	Mann-Whitney U test	U = 80.00000	Two-tailed	P = 0.4336; ns	Yes; P = 0.5444; ns
					cKO				7943.099 ± 440.9978						No; P = 0.0490; *
	A	Open-field test	Distance moved (m)	10	WT	11-13 weeks	WT = 23<br>KO = 22	Male	46.21747		Repeated measures of two-way ANOVA; Bonferroni's multiple comparison test	Interaction<br>F(5, 215) = 3.056932, P = 0.0109 ; *<br><br>Genotype<br>F(1, 43) = 39.81172, P < 0.0001 ; ***<br><br>Time<br>F(5, 215) = 159.7164, P < 0.0001 ; ***		P < 0.001; ***	
					KO				34.43741						
				20	WT				34.71045					P < 0.001; ***	
					KO				23.98065						
				30	WT				31.36455					P < 0.001; ***	
					KO				19.77695						
				40	WT				28.48259					P < 0.001; ***	
					KO				17.80916						
				50	WT				24.68907					P < 0.01; **	
					KO				17.607						
				60	WT				22.58289					P < 0.01; **	
					KO				15.35223						
			Total distance moved (m)	WT					18804.87 ± 823.3651	O	Student's t-test	t(43) = 6.309	Two-tailed	P < 0.0001 ; ***	Yes; P = 0.2394; ns
				KO					12896.52 ± 418.6214						Yes; P = 0.8752; ns
				2	WT				43.36399					P < 0.001; ***	
					KO				26.68781						
				4	WT				23.4012					P > 0.05; ns	
					KO				21.0029						
				6	WT				19.78495					P > 0.05; ns	
					KO				18.10538						
				8	WT				13.4726					P > 0.05; ns	
					KO				17.66133						
				10	WT				10.6592					P > 0.05; ns	
					KO				9.192524						
				12	WT				14.65945					P > 0.05; ns	
					KO				14.35176						
				14	WT				5.70035					P > 0.05; ns	
					KO				7.766239						
				16	WT				3.63815					P > 0.05; ns	
					KO				5.017334						
				18	WT				2.6577					P > 0.05; ns	
					KO				1.808667						
				20	WT				3.0988					P > 0.05; ns	
					KO				2.301286						
				22	WT				3.7996					P > 0.05; ns	
					KO				3.387333						

6	B	Long-term (96 hr) locomotor activity	Distance moved (m)	24	WT	9-14 weeks	WT = 20<br>KO = 21	Male	3.73705		Repeated measures of two-way ANOVA; Bonferroni's multiple comparison test	Interaction<br>F(47, 1833) = 2.910928, P < 0.0001; ***<br><br>Genotype<br>F(1, 39) = 0.8188657, P = 0.3711 ; ns<br><br>Time<br>F(47, 1833) = 60.50089, P < 0.0001; ***		P > 0.05; ns	
					KO				2.403571						
				26	WT				19.36845					P > 0.05; ns	
					KO				14.78681						
				28	WT				22.98195					P > 0.05; ns	
					KO				17.14157						
				30	WT				15.5836					P > 0.05; ns	
					KO				18.61228						
				32	WT				13.15085					P > 0.05; ns	
					KO				13.66267						
				34	WT				11.0785					P > 0.05; ns	
					KO				10.42595						
				36	WT				11.4361					P > 0.05; ns	
					KO				13.73681						
				38	WT				3.73725					P > 0.05; ns	
					KO				4.787477						
				40	WT				4.0423					P > 0.05; ns	
					KO				4.350095						
				42	WT				2.7999					P > 0.05; ns	
					KO				2.567286						
				44	WT				3.0446					P > 0.05; ns	
					KO				2.729						
				46	WT				4.84315					P > 0.05; ns	
					KO				2.395238						
				48	WT				6.56065					P > 0.05; ns	
					KO				3.688191						
				50	WT				21.9538					P > 0.05; ns	
					KO				15.93752						
				52	WT				22.2018					P > 0.05; ns	
					KO				17.10329						
				54	WT				18.60465					P > 0.05; ns	
					KO				16.76214						
				56	WT				14.92115					P > 0.05; ns	
					KO				16.23405						
				58	WT				11.03155					P > 0.05; ns	
					KO				12.33733						
				60	WT				12.88435					P > 0.05; ns	
					KO				13.37781						
				62	WT				4.2277					P > 0.05; ns	
					KO				5.556905						
				64	WT				4.1882					P > 0.05; ns	
					KO				3.257238						
				66	WT				2.5487					P > 0.05; ns	
					KO				3.87219						
				68	WT				3.574					P > 0.05; ns	
					KO				2.412381						
				70	WT				4.7479					P > 0.05; ns	
					KO				3.566667						
				72	WT				5.0131					P > 0.05; ns	
					KO				5.649667						
				74	WT				20.68365					P > 0.05; ns	
					KO				17.3539						
				76	WT				23.61865					P > 0.05; ns	
					KO				17.43781						
				78	WT				19.64345					P > 0.05; ns	
					KO				18.93771						
				80	WT				14.7552					P > 0.05; ns	
					KO				13.53305						
				82	WT				10.50255					P > 0.05; ns	
					KO				14.07171						
				84	WT				14.03875					P > 0.05; ns	
					KO				12.67876						
				86	WT				3.793399					P > 0.05; ns	
					KO				4.646333						
				88	WT				4.2938					P > 0.05; ns	
					KO				1.628238						
				90	WT				3.67975					P > 0.05; ns	
					KO				2.040524						
				92	WT				5.20055					P > 0.05; ns	
					KO				2.329048						
				94	WT				5.58125					P > 0.05; ns	
					KO				5.033286						
				96	WT				6.543301					P > 0.05; ns	
					KO				6.538476						
			Total distance moved (m)	First 6hrs	WT				86.55015 ± 6.752912	O	Student's t-test	t(39) = 2.325158	Two-tailed	P = 0.0254; *	Yes; P = 0.7015; ns
					KO				65.79610 ± 5.875874						Yes; P = 0.7631; ns
				Last 72hrs	WT				376.9 ± 32.47	O	Student's t-test	t(39) = 0.7520	Two-tailed	P = 0.4565; ns	Yes; P = 0.7508; ns
					KO				341.2 ± 34.46						Yes; P = 0.6181; ns
	C	Open-field test	Distance moved (m)	10	WT	8-9 weeks	WT = 18<br>cKO = 15	Male	42.9333		Repeated measures of two-way ANOVA; Bonferroni's multiple comparison test	Interaction<br>F(5, 155) = 2.32509, P = 0.0454.; *<br><br>Genotype<br>F(1, 31) = 14.6316, P = 0.0006; ***<br><br>Time<br>F(5, 155) = 167.0022, P < 0.0001; ***		P < 0.001; ***	
					cKO				33.31202						
				20	WT				29.74141					P < 0.05; *	
					cKO				23.04503						
				30	WT				24.04476					P < 0.05; *	
					cKO				17.90403						
				40	WT				21.01366					P < 0.05; *	
					cKO				14.3757						
				50	WT				16.28053					P > 0.05; ns	
					cKO				13.36621						
				60	WT				17.53495					P < 0.05; *	
					cKO				11.21964						
			Total distance moved (m)	WT					151.5511 ± 7.54495	O	Student's t-test	t(31) = 3.825251	Two-tailed	P = 0.0006; ***	Yes; P = 0.5376; ns
				cKO					113.2233 ± 6.18914						Yes; P = 0.6089; ns
	D	Long-term (92 hr) locomotor activity	Distance moved (m)	2	WT	12-14 weeks	WT = 15<br>cKO = 13	Male	28.2778		Repeated measures of two-way ANOVA; Bonferroni's multiple comparison test	Interaction<br>F(45, 1170) = 1.949454, P = 0.0002.; ***<br><br>Genotype<br>F(1, 26) = 0.1112433, P = 0.7414; ns<br><br>Time<br>F(45, 1170) = 38.27623, P < 0.0001; ***		P > 0.05; ns	
					cKO				23.70577						
				4	WT				24.9736					P > 0.05; ns	
					cKO				20.14523						
				6	WT				21.6296					P > 0.05; ns	
					cKO				15.36331						
				8	WT				15.20213					P > 0.05; ns	
					cKO				13.82415						
				10	WT				9.429867					P > 0.05; ns	
					cKO				5.937462						
				12	WT				15.8082					P > 0.05; ns	
					cKO				13.81646						
				14	WT				7.101533					P > 0.05; ns	
					cKO				10.88977						
				16	WT				3.4812					P > 0.05; ns	
					cKO				3.210077						
				18	WT				3.0448					P > 0.05; ns	
					cKO				4.541615						
				20	WT				2.8274					P > 0.05; ns	
					cKO				3.973						
				22	WT				4.6272					P > 0.05; ns	
					cKO				4.127692						
				24	WT				4.249733					P > 0.05; ns	
					cKO				6.192153						
				26	WT				7.263134					P > 0.05; ns	
					cKO				13.94754						
				28	WT				19.5348					P > 0.05; ns	
					cKO				18.15454						
				30	WT				18.55367					P > 0.05; ns	
					cKO				15.31023						
				32	WT				13.57713					P > 0.05; ns	
					cKO				10.81723						
				34	WT				9.441867					P > 0.05; ns	
					cKO				11.71115						
				36	WT				14.74653					P > 0.05; ns	
					cKO				16.41585						
				38	WT				4.8132					P > 0.05; ns	
					cKO				6.365154						
				40	WT				3.686134					P > 0.05; ns	
					cKO				2.618615						
				42	WT				2.603534					P > 0.05; ns	
					cKO				2.443154						
				44	WT				2.945467					P > 0.05; ns	
					cKO				4.390461						
				46	WT				3.9268					P > 0.05; ns	
					cKO				5.147308						
				48	WT				4.5042					P > 0.05; ns	
					cKO				5.776154						
				50	WT				11.8068					P > 0.05; ns	
					cKO				17.51446						
				52	WT				19.65087					P > 0.05; ns	
					cKO				16.21923						
				54	WT				16.14287					P > 0.05; ns	
					cKO				12.79554						
				56	WT				11.7064					P > 0.05; ns	
					cKO				12.74992						
				58	WT				9.121401					P > 0.05; ns	
					cKO				9.850923						
				60	WT				14.81747					P > 0.05; ns	
					cKO				16.13515						
				62	WT				5.112333					P > 0.05; ns	
					cKO				2.943923						
				64	WT				3.465867					P > 0.05; ns	
					cKO				1.804231						
				66	WT				3.970334					P > 0.05; ns	
					cKO				3.505077						
				68	WT				3.305467					P > 0.05; ns	

				[illegible]	cKO				8.503308					P > 0.05; ns	
				70	WT				4.3922					P > 0.05; ns	
					cKO				5.680154						
				72	WT				4.019667					P > 0.05; ns	
					cKO				5.162231						
				74	WT				13.0278					P > 0.05; ns	
					cKO				18.31015						
				76	WT				16.37993					P > 0.05; ns	
					cKO				15.58631						
				78	WT				13.76387					P > 0.05; ns	
					cKO				15.36546						
				80	WT				12.092					P > 0.05; ns	
					cKO				15.89469						
				82	WT				9.531668					P > 0.05; ns	
					cKO				10.787						
				84	WT				12.5404					P > 0.05; ns	
					cKO				14.04592						
				86	WT				4.631999					P > 0.05; ns	
					cKO				3.249769						
				88	WT				1.971333					P > 0.05; ns	
					cKO				3.061154						
				90	WT				2.976133					P > 0.05; ns	
					cKO				2.159769						
				92	WT				4.036734					P > 0.05; ns	
					cKO				4.012						
			Total distance moved (m)	First 6hrs	WT				74.881 ± 3.861452	O	Student's t-test	t(26) = 2.649986	Two-tailed	P = 0.0135; *	Yes; P = 0.8231; ns
					cKO				59.21431 ± 4.527318						Yes; P = 0.4284; ns
				Last 68hrs	WT				304.06 ± 15.19005	O	Student's t-test	t(26) = 1.002142	Two-tailed	P = 0.3255; ns	Yes; P = 0.5010; ns
					cKO				328.4338 ± 19.39085						Yes; P = 0.8935; ns
7	A	Open-field test	Time in center (%)	WT		11-13 weeks	WT = 23<br>KO = 22	Male	21.51334 ± 1.602101	O	Student's t-test	t(43) = 0.9524121	Two-tailed	P = 0.3462 ; ns	Yes; P = 0.7564; ns
				KO					19.44934 ± 1.450492						Yes; P = 0.6812; ns
	B	Elevated plus-maze test	Time in arms (s)	WT	Open	13-21 weeks	WT = 22<br>KO = 20	Male	60.18313 ± 8.862339	O	Paired t-test	t(21) = 14.30209	Two-tailed	P < 0.0001; ***	Yes; P = 0.4961; ns
					Closed				325.9707 ± 10.21304						Yes; P = 0.9018; ns
				KO	Open				99.10365 ± 10.97282	O	Paired t-test	t(19) = 8.184796	Two-tailed	P < 0.0001; ***	Yes; P = 0.1121; ns
					Closed				299.4698 ± 15.42351						Yes; P = 0.0996; ns
			Time in open arms (%)	WT					12.53815 ± 1.846321	O	Student's t-test	t(40) = 2.781636	Two-tailed	P = 0.0082; **	Yes; P = 0.4961; ns
				KO					20.6466 ± 2.286003						Yes; P = 0.1121; ns
	C	Light-dark test	Time in light chamber (%)	WT		14-21 weeks	WT = 23<br>KO = 20	Male	24.2682 ± 2.066739	O	Student's t-test	t(41) = 2.567099	Two-tailed	P = 0.0140; *	Yes; P = 0.113; ns
				KO					16.5145 ± 2.200488						Yes; P = 0.1944; ns
			Number of transitions	WT					33.82609 ± 2.614683	O	Student's t-test	t(41) = 3.127205	Two-tailed	P = 0.0032; **	Yes; P = 0.6414; ns
				KO					22.45 ± 2.484134						Yes; P = 0.4084; ns
	D	Open-field test	Time in center (%)	WT		8-9 weeks	WT = 18<br>cKO = 15	Male	17.277 ± 1.419751	O	Student's t-test	t(31) =2.777548	Two-tailed	P = 0.0092; **	Yes; P = 0.5961; ns
				cKO					11.90276 ± 1.258791						Yes; P = 0.2884; ns
	E	Elevated plus-maze test	Time in arms (s)	WT	Open	8-10 weeks	WT = 19<br>cKO = 15	Male	81.46753 ± 11.47724	O	Paired t-test	t(18) = 6.381678	Two-tailed	P < 0.0001; ***	Yes; P = 0.3020; ns
					Closed				250.6313 ± 16.09347						Yes; P = 0.7081; ns
				KO	Open				74.98675 ± 11.71309	O	Paired t-test	t(14) = 7.372885	Two-tailed	P < 0.0001; ***	Yes; P = 0.0923; ns
					Closed				286.9499 ± 17.95637						Yes; P = 0.3824; ns
			Time in open arms (%)	WT					16.9724 ± 2.391091	O	Student's t-test	t(32) =0.3905488	Two-tailed	P = 0.6987; ns	Yes; P = 0.3020; ns
				cKO					15.62224 ± 2.440228						Yes; P = 0.0923; ns
	F	Light-dark test	Time in light chamber (%)	WT		11-20 weeks	WT = 19<br>cKO = 15	Male	28.14627 ± 1.959742	X	Mann-Whitney U test	U = 50.00000	Two-tailed	P = 0.0014; **	Yes; P = 0.4661; ns
				cKO					18.22451 ± 2.179838						No; P = 0.0280; *
			Number of transitions	WT					30.73684 ± 1.861965	O	Student's t-test	t(32) = 3.757887	Two-tailed	P = 0.0007; ***	Yes; P = 0.0896; ns
				cKO					20 ± 2.186539						Yes; P = 0.2261; ns
